# The ZmCOP1s–ZmCOL3 Module Enhances Late Flowering, Grain Yield and Grain Quality in Maize

**DOI:** 10.1111/pbi.70643

**Published:** 2026-03-18

**Authors:** Shuling Yang, Yanpei Zhang, Lianhua Cui, Weimin Zhan, Shizhan Chen, Yu Zhang, Haolei Han, Linhu Song, Longchao Du, Kangni Zhang, Huan Liu, Shaoci Wang, Yong Shi, Jihua Tang, Xiangguo Liu, Jianping Yang

**Affiliations:** ^1^ State Key Laboratory of Wheat and Maize Crop Science, Collaborative Innovation Center of Henan Grain Crops, College of Agronomy Henan Agricultural University Zhengzhou China; ^2^ Management Committee of the Caina National Modern Agricultural Demonstration Zone Qushui County China; ^3^ Institute of Agricultural Biotechnology, Jilin Academy of Agricultural Sciences (Northeast Innovation Center of Agricultural Science and Technology in China) Changchun China

**Keywords:** flowering time, grain yield, nutritional quality, *Zea mays*, ZmCOP1s‐ZmCOL3 module

## Abstract

Flowering time is a key determinant of yield and regional adaptation in crops and is largely controlled by light signalling. In this study, we identified two maize orthologs of *Arabidopsis* CONSTITUTIVE PHOTOMORPHOGENIC 1 (COP1), designated ZmCOP1a and ZmCOP1b, which activate light signalling and reduce plant height. Loss‐of‐function mutants of *ZmCOP1a* and *ZmCOP1b* flowered early, whereas overexpression of either gene significantly delayed flowering under both long‐ and short‐day conditions. Mechanistically, both ZmCOP1a and ZmCOP1b interacted with and stabilised the C2C2‐CO‐like transcription factor ZmCOL3. Together, they upregulated key repressors of flowering (*ZmPRR37*, *ZmPRR95*, *ZmCCA1* and *ZmCCT9*), leading to suppression of florigen genes (*ZCN7*, *ZCN8* and *ZCN12*) and floral meristem identity genes (*ZMM3*, *ZMM4* and *ZMM15*). Field trials demonstrated that overexpression of *ZmCOP1a* or *ZmCOP1b* reduced starch content but increased protein levels, kernel numbers and kernel length, width and weight. In contrast, overexpression of *ZmCOL3* decreased both starch and protein content. Collectively, our results demonstrate that the ZmCOP1s–ZmCOL3 module is a key regulator of flowering time and source–sink reallocation, providing valuable genetic targets for breeding high‐yield, high‐quality maize with optimised flowering time and enhanced carbon and nitrogen allocation.

## 1 Introduction

Maize ( *Zea mays* ) is the most widely produced cereal worldwide: annual yields surpass 1 billion metric tons and represent approximately 40% of global grain output (Liu et al. 2020; Zhao et al. 2023). As the demand for food, feed and industrial uses continues to rise, improving maize yield has become a major goal of crop breeding programs (Li and Wang 2009; Li et al. 2016). Domesticated from the short‐day (SD) flowering teosinte ( *Zea mays* ssp. *parviglumis* ) in Mexico, the successful adaptation of modern maize to a broad range of photoperiods has underpinned its latitudinal spread and global cultivation (Liu et al. 2015). Despite this adaptability, precise control over flowering time remains crucial for maximising yield potential in target environments (Buckler et al. 2009; Niu et al. 2024).

The transition to flowering in maize is coordinately regulated by five major signalling pathways: light perception, photoperiod/circadian regulation, the autonomous pathway, hormonal signalling and the age‐dependent pathway. These pathways converge on key floral integrators such as *FLOWERING LOCUS T‐like* (*ZCN*) genes and MADS‐box transcription factors (*ZMMs*) (Choquette et al. 2023). Phytochromes (PHYA/B/C) and cryptochromes (CRY) act as primary photoreceptors, initiating a downstream cascade of photoreceptor, circadian and florigen gene activity that precisely regulates flowering time (Chen, Fan, et al. 2024; Li et al. 2020; Zhao et al. 2014). The circadian clock is the central regulator of this network. In the morning loop, pseudo‐response regulators ZmPRR37/73 function as flowering repressors (Yang et al. 2023). During the evening phase, the evening complex (ZmELF3, ZmELF4 and ZmLUX) antagonises the inhibitory effects of ZmPRR37/73, CONSTANS‐LIKE proteins ZmCCT9/10 and the C2C2‐CO‐like transcription factor ZmCOL3. This antagonism releases repression of *CENTRORADIALIS* genes (*ZCN7/8/12*), thereby promoting flowering (Huang et al. 2018; Zhao et al. 2023). Conversely, ZmCCT and ZmCOL3 delay heading by repressing florigen genes (*ZmFT* and *ZCN8*) (Jin et al. 2018; Yang et al. 2013). The core circadian oscillator components *GIGANTEA1/2* (*ZmGI1/2*) negatively regulate *ZCN8* expression (Li, Gao, et al. 2023), whereas the F‐box protein ZmFKF1 acts as a flowering promoter (Chen, Gao, et al. 2024). ZmCCA1 and ZmTOC1 form a reciprocal feedback loop to maintain circadian rhythmicity (Bendix et al. 2013). Further fine‐tuning of flowering time involves multiple regulators, including *Delayed Flowering1* (*DLF1*) (Sun et al. 2020), nuclear factors *ZmNF‐YA3*/*C2* (Su et al. 2021), *EARLY FLOWERING6* (*ZmELF6*) (Su et al. 2024) and the AAA + ATPase *ZmOM66* (Du et al. 2025). Within the autonomous pathway, the floral regulator *INDETERMINATE1* (ID1) mediates phloem transport of *ZCN8* mRNA to initiate reproductive transition (Colasanti et al. 1998). Genetic evidence from *d8* mutants has demonstrated that gibberellin signalling is a critical hormonal checkpoint for flowering (Thornsberry et al. 2001). Ultimately, the age‐dependent miR156–SPL–miR172–AP2 module triggers floral induction through activation of *ZMM3/4* (Yang et al. 2023).

CONSTITUTIVE PHOTOMORPHOGENIC 1 (COP1), which encodes a RING‐type E3 ubiquitin ligase, acts as a central regulator of light signalling pathways that govern diverse developmental processes, including seed germination, seedling de‐etiolation, photomorphogenesis and flowering time control (Han et al. 2020; Lau and Deng 2012). In *Arabidopsis*, COP1 regulates flowering through multiple molecular mechanisms, including direct regulation of CONSTANS (CO) protein stability, circadian regulation and substrate‐dependent regulation. Under long‐day (LD) conditions, light‐mediated suppression of COP1 activity permits CO accumulation, leading to *FLOWERING LOCUS T* (FT) activation and floral induction. Conversely, under SD conditions, sustained COP1 activity promotes CO degradation, thereby inhibiting flowering. Notably, blue light perception through cryptochrome 2 (CRY2) induces CRY2–SPA1 complex formation, which sequesters COP1 and consequently stabilises CO protein to enhance *FT* expression (Zuo et al. 2011). COP1 physically interacts with *EARLY FLOWERING 3* (ELF3) to mediate ubiquitin‐dependent degradation of *GIGANTEA* (GI), thereby modulating circadian rhythm amplitude and phase. This post‐translational regulation ultimately affects *CO* transcript levels and flowering time (Yu et al. 2008). The COP1–SPA E3 ligase complex targets *CONSTANS‐like 12* (COL12) for degradation under dark conditions; upon light exposure, stabilised COL12 protein accumulates and suppresses *FT* expression, resulting in delayed flowering (Ordoñez‐Herrera et al. 2018; Zhou et al. 2024). Although the molecular functions of COP1 have been extensively characterised in *Arabidopsis*, its biological roles and mechanisms in maize remain entirely unknown.

In this study, we characterised two maize COP1 orthologs, ZmCOP1a and ZmCOP1b, which consistently delayed flowering time under both LD and SD photoperiods. Protein interaction assays demonstrated direct binding between ZmCOP1a/b and the floral regulator ZmCOL3, leading to transcriptional activation of the key flowering genes (*ZCN7*/*8*/*12* and *ZMM3*/*4*/*15*). Strikingly, transgenic overexpression of ZmCOP1a/b enhanced kernel nutritional quality by reducing starch while increasing protein content, whereas ZmCOL3 overexpression decreased both components. Our results establish the ZmCOP1s–COL3 module as a dual‐function regulator capable of coordinately improving both yield and grain quality in maize.

## 2 Results

### 2.1 Phylogenetic Relationships and Expression Profiles of *ZmCOP1a* and *ZmCOP1b*

**FIGURE 1:**
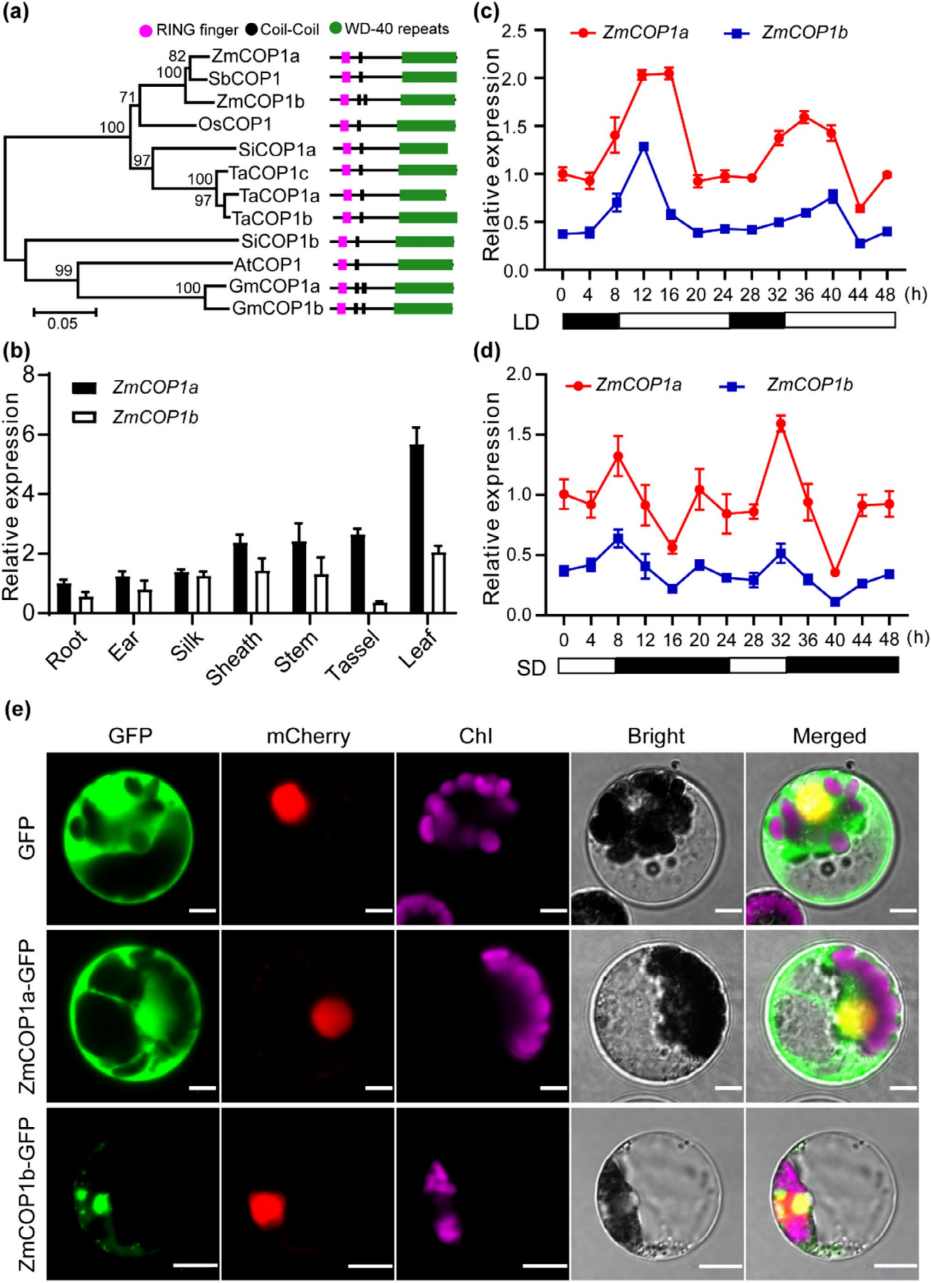
Phylogenetic analysis, expression profiles and subcellular localization of ZmCOP1a and ZmCOP1b. (a) Phylogenetic analysis and domain architecture of COP1 homologues in maize and selected plant species. Accession numbers: ZmCOP1a ( *Zea mays* , Zm00001d018207), ZmCOP1b ( *Zea mays* , Zm00001d052138), SbCOP1 ( *Sorghum bicolor* , KXG31183), SiCOP1a ( *Setaria italica* , KQK01702), SiCOP1b ( *Setaria italica* , SIN_1026932.t), OsCOP1 ( *Oryza sativa* , Os02g0771100), TaCOP1a ( *Triticum aestivum* , TraesCS6A02G326100), TaCOP1b ( *Triticum aestivum* , TraesCS6D02G305800), TaCOP1c ( *Triticum aestivum* , TraesCS6B02G356400), GmCOP1a ( *Glycine max* , KRH14801), GmCOP1b ( *Glycine max* , KRH73341), AtCOP1 ( *Arabidopsis thaliana* , AT2G32950). (b) Tissue‐specific expression patterns of *ZmCOP1a* and *ZmCOP1b*. Expression in roots was set as the reference. Values represent means ± standard error (SE) (*n* = 3). (c, d) Diurnal expression profiles of *ZmCOP1a* and *ZmCOP1b* under long‐day (LD) (c) and short‐day (SD) (d) conditions. Expression at 0 h was used as the reference. Black and white bars indicate dark and light periods, respectively. Data are means ± SE (*n* = 4). (e) Subcellular localization of ZmCOP1a–green fluorescent protein (GFP) and ZmCOP1b–GFP in maize protoplasts. Chlorophyll autofluorescence (Chl) marks chloroplasts; mCherry was used as a nuclear marker. Scale bar = 20 μm. (See also Figure S1).

Using the amino acid sequence of *Arabidopsis* COP1, we conducted a BLAST search of the UniProt database (https://www.uniprot.org) and identified two maize homologues: ZmCOP1a (*Zm00001d018207*) and ZmCOP1b (*Zm00001d052138*). Phylogenetic analysis showed that both maize proteins clustered with COP1 homologues from other grasses and were most closely related to sorghum COP1. Each protein contains characteristic conserved domains, including a RING finger (R), a coiled‐coil (C) and WD40 repeats (Figure 1a). Expression analysis across various tissues revealed ubiquitous expression of *ZmCOP1a* and *ZmCOP1b*, with the highest transcript levels detected in leaves. Notably, *ZmCOP1a* expression was significantly higher than that of *ZmCOP1b* in leaves (Figure 1b). Circadian analysis showed that both genes displayed similar diurnal oscillation patterns under both LD and SD conditions, with *ZmCOP1a* exhibiting higher amplitude fluctuations (Figure 1c,d). Subcellular localization assays demonstrated that ZmCOP1a–green fluorescent protein (GFP), ZmCOP1b–GFP and the truncated variants ZmCOP1b‐ΔR–GFP and ZmCOP1b‐ΔCC–GFP were present in both the nucleus and cytoplasm. Notably, ZmCOP1a–GFP exhibited stronger nuclear fluorescence signals (Figure 1e; Figure S1). These findings suggest that both ZmCOP1a and ZmCOP1b respond to photoperiodic changes and play important roles in regulating maize flowering time.

### 2.2 Both ZmCOP1a and ZmCOP1b Activate Light Signalling and Reduce Plant Height

COP1 has been shown to promote seedling etiolation In *Arabidopsis* (Deng et al. 1992). To investigate the functional roles of ZmCOP1a and ZmCOP1b in skotomorphogenesis, we generated binary vectors carrying each gene (Figure S2a) and expressed them in the *Arabidopsis cop1‐4* mutant (Yang et al. 2005). When grown in darkness (Dk), transgenic lines of *ZmCOP1a*/*cop1‐4* (#2 and #6) and *ZmCOP1b*/*cop1‐4* (#8 and #11) displayed phenotypes comparable to wild‐type (WT) *Arabidopsis* (Col‐0). Under white light (W) conditions, these transgenic lines exhibited significantly elongated hypocotyls (Figure S2b; Figure S3a,b). The chlorophyll and anthocyanin contents, as well as the transcript levels of *CAB3* (*Chlorophyll a/b‐binding protein 3*) and *CHS* (*Chalcone Synthase*), in these transgenic lines were comparable to those in WT plants and significantly lower than those in the *cop1‐4* mutant (Figure S3c,d). Furthermore, under both LD and SD conditions, plant height was increased in the complemented lines compared with the WT lines (Figure S3b,e,f). These results demonstrate that both ZmCOP1a and ZmCOP1b are functional orthologs capable of promoting etiolation in *Arabidopsis* seedlings.

**FIGURE 2:**
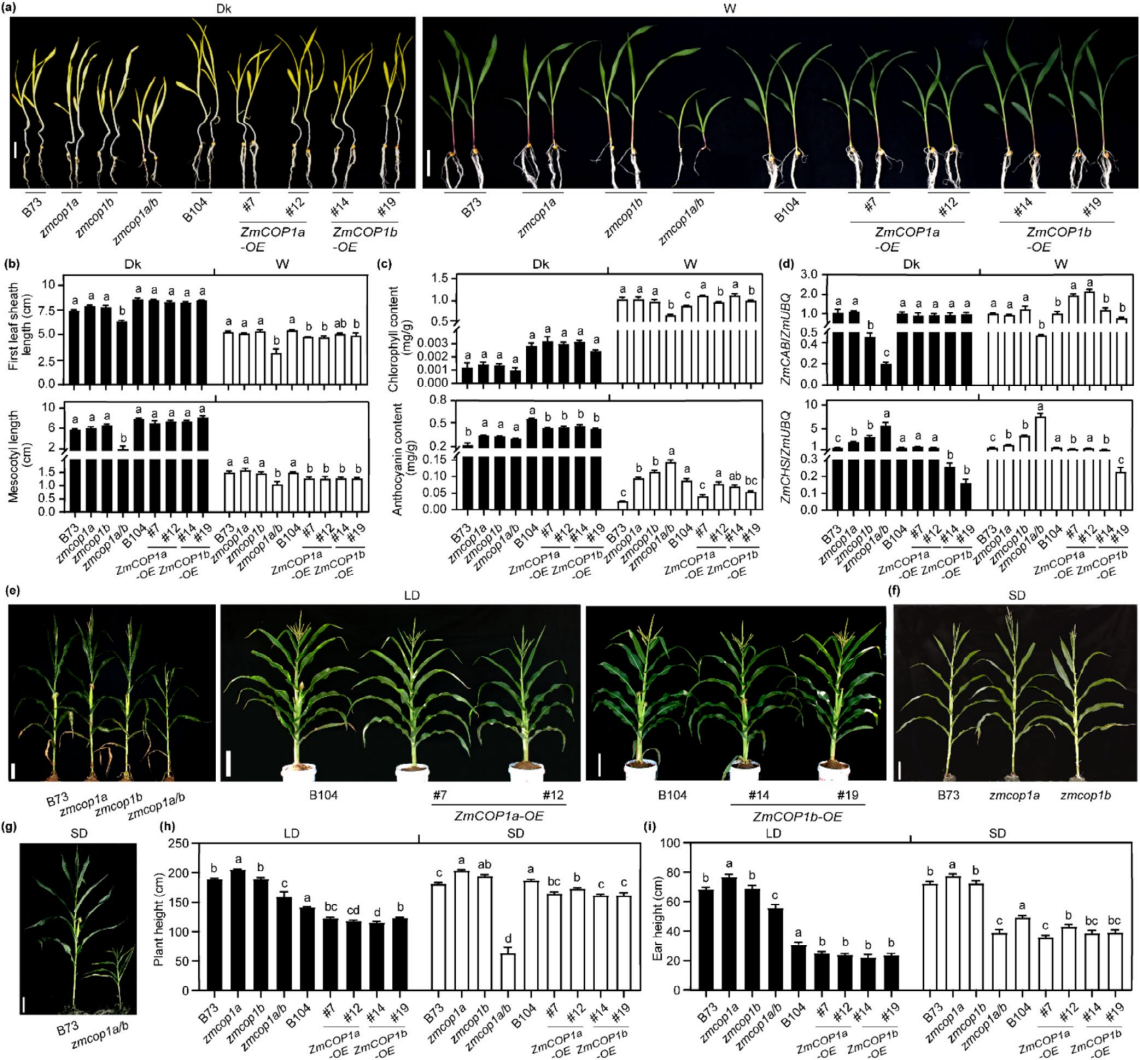
Both ZmCOP1a and ZmCOP1b negatively regulate plant height in maize. (a) Morphology of inbred line B73 (wild‐type line, WT1), inbred line B104 (transgenic background, WT2), single mutants, double mutant and overexpression lines of *ZmCOP1a* and *ZmCOP1b* grown in darkness (Dk) or under white light (W, 70 μmol·m^−2^·s^−1^) at 26°C for 10 days. Scale bar = 5 cm. (b) First‐leaf sheath length and mesocotyl length measurements corresponding to (a). *n* ≥ 10. (c) Chlorophyll and anthocyanin contents corresponding to (a). (d) Relative transcriptional levels of *ZmCAB* and *ZmCHS* corresponding to (a). (e–g) Adult plant morphologies of WT1, WT2, single mutant, double mutant and overexpression lines of *ZmCOP1a* and *ZmCOP1b* under long‐day (LD) conditions in Henan Province for 60 days and short‐day (SD) conditions in Hainan Province for 65 days. Scale bar = 20 cm. (h, i) Plant height (h) and ear height (i) measurements for plants grown under LD and SD conditions. Sample sizes: Single mutants, *n* ≥ 17; double mutants, *n* ≥ 4; overexpression lines, *n* ≥ 10. (See also Figures S2–S6).

To investigate whether ZmCOP1a and ZmCOP1b activate light signalling in maize, we characterised ethyl methanesulfonate (EMS)‐induced single mutants (*zmcop1a*, containing an R143‐to‐stop mutation; *zmcop1b*, containing a Q299‐to‐stop mutation; Figure S2c–e), a double mutant *zmcop1a*/*b* generated by crossing the single mutants, and two overexpression (OE) lines, *ZmCOP1a‐OE* (#7, #12) and *ZmCOP1b‐OE* (#14, #19) in the B104 inbred background (Figure S2a,b), under various light conditions. Phenotypic analysis revealed that the first leaf sheath and mesocotyl lengths of the *zmcop1a* and *zmcop1b* single mutants were not significantly altered compared with those of WT plants when grown under Dk, W, far‐red light (FR), or blue light (B) conditions. By contrast, the *zmcop1a*/*b* double mutants showed significant reductions in first‐leaf sheath length (by 85.62%, 59.87%, 76.67% and 61.39%) and mesocotyl length (by 51.0%, 70.0%, 72.0% and 61.30%) under Dk, W, FR and B conditions, respectively. Under red light (R) conditions, neither first‐leaf sheath length nor mesocotyl length was significantly altered in the double mutant. The *zmcop1a* and *zmcop1b* single mutants exhibited increases of 9.4% and 12.6%, respectively, in first‐leaf sheath length compared with the B73 WT line. Both the *ZmCOP1a‐OE* and *ZmCOP1b‐OE* overexpression lines exhibited slight reductions in first‐leaf sheath and mesocotyl length across multiple light conditions (Figure 2a,b; Figure S4a–d).

To further characterise these phenotypes at the physiological level, we quantified chlorophyll and anthocyanin accumulation under various light conditions. Under R and W conditions, the *zmcop1a*/*b* double mutant showed reduced chlorophyll content and downregulated expression of *ZmCAB*. In contrast, the *ZmCOP1a‐OE* and *ZmCOP1b‐OE* overexpression lines accumulated approximately 1.2 times more chlorophyll than the B104 WT control. No significant differences in chlorophyll content were detected under Dk, FR, or B conditions (Figure 2a,c,d; Figure S4a,c,e). Under various light conditions, anthocyanin content was significantly increased in the *zmcop1a*, *zmcop1b* and *zmcop1a*/*b* double mutants, consistent with the upregulation of *ZmCHS* expression. Conversely, the *ZmCOP1a‐OE* and *ZmCOP1b‐OE* overexpression lines exhibited decreased anthocyanin accumulation and a slight reduction in *ZmCHS* transcript levels (Figure 2c,d; Figure S4f). These results demonstrate that ZmCOP1a and ZmCOP1b function as positive regulators of skotomorphogenesis, mediating light signalling to suppress mesocotyl and first‐leaf sheath elongation, promote chlorophyll biosynthesis and inhibit anthocyanin accumulation in maize seedlings.

Plant height and ear height were significantly increased in the *zmcop1a* mutant under both LD and SD field conditions. The *zmcop1b* mutant also exhibited increased plant height under SD conditions, whereas the *ZmCOP1a‐OE* and *ZmCOP1b‐OE* overexpression lines showed reduced plant height, ear height and internode lengths compared with WT plants (Figure 2e,f,h,i; Figure S5). The *zmcop1a*/*b* double mutant displayed a pronounced dwarf phenotype (Figure 2g,h). Furthermore, *ZmCOP1a*/*b* promoted the accumulation of ZmHY5 protein and upregulated the expression of *ZmGA2ox10*, a key gibberellin catabolism enzyme (Figure S6). These results support a model in which the *ZmCRY1b–ZmCOP1*/*SPA1s–ZmHY5–ZmGA2ox10* module negatively regulates plant height (Chen, Fan, et al. 2024). Collectively, these findings demonstrate that ZmCOP1a and ZmCOP1b are central regulators of both plant and ear height in maize.

### 2.3 ZmCOP1a and ZmCOP1b Delay Flowering Under Both LD and SD Conditions

COP1 has been shown to repress flowering in *Arabidopsis* (Liu et al. 2008). Consistent with a role in photoperiodic response, circadian analysis indicated that both ZmCOP1a and ZmCOP1b are responsive to day length (Figure 1c,d). Compared with the *cop1‐4* mutant, the *ZmCOP1a*/*cop1‐4* and *ZmCOP1b*/*cop1‐4* transgenic lines flowered significantly later under both photoperiod regimes. Under LD conditions, flowering time was delayed by 4.6 and 4.0 days, accompanied by increases of 2.8 and 3.9 rosette leaves, respectively. Under SD conditions, the delay was more pronounced, with flowering occurring approximately 35.1 and 56.0 days later and increases of 12.4 and 8.8 rosette leaves, respectively (Figure S7a–d). Moreover, transcript levels of key flowering‐promoting genes [*FT*, *CO* and *SUPPRESSOR OF OVEREXPRESSION OF CONSTANS 1* (*SOC1*)] were significantly reduced in these transgenic lines compared with both the *cop1‐4* mutant and WT lines (Figure S7e). Thus, ZmCOP1a and ZmCOP1b inhibit flowering in *Arabidopsis*.

**FIGURE 3:**
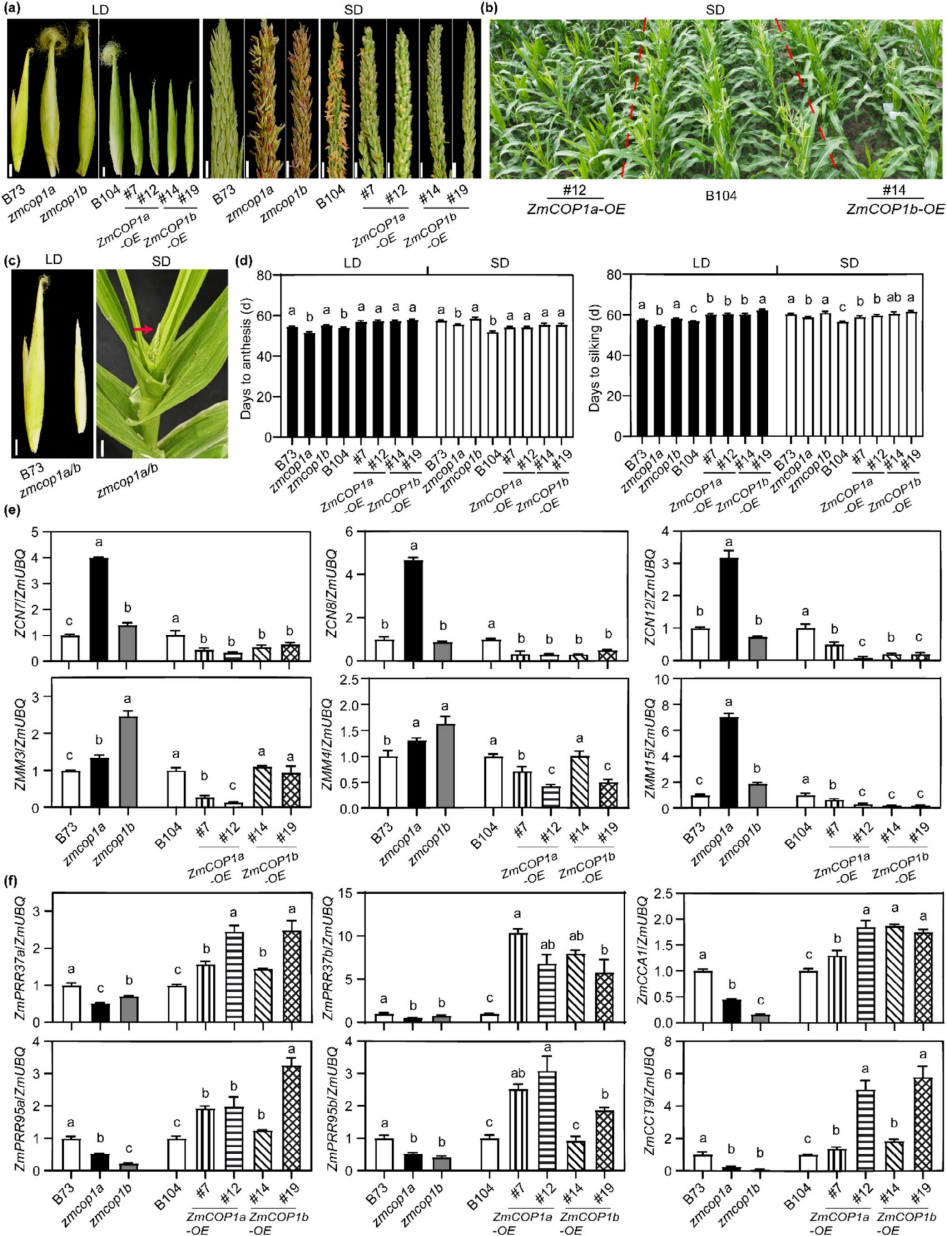
Both ZmCOP1a and ZmCOP1b delay flowering in maize. (a) Tassel and ear phenotypes of WT1, WT2, single mutant, double mutant and overexpression lines of *ZmCOP1a* and *ZmCOP1b*. Scale bars: Tassel = 1 cm, ear = 2 cm. (b) Phenotypes of the WT2 and overexpression lines of *ZmCOP1a* and *ZmCOP1b* at anthesis under SD conditions. (c) Tassel and ear phenotypes of the *zmcop1a*/*b* double mutant under LD and SD conditions. Red arrow indicates tassel. Scale bars: Tassel = 5 mm, ear = 2 cm. (d) Flowering time (days to anthesis) and silking time (days to silking) of WT1, WT2, single mutant and overexpression lines of *ZmCOP1a* and *ZmCOP1b* under LD and SD conditions. *n* ≥ 15. (e) Transcriptional abundances of florigen genes (*ZCN7*, *ZCN8* and *ZCN12*) and floral meristem identity genes (*ZMM3*, *ZMM4*, anf *ZMM15*) in unpollinated tassels of WT1, WT2, single mutant and overexpression lines of *ZmCOP1a* and *ZmCOP1b. Seedlings were grown* under LD conditions for 53 days after germination (DAG). Data are means ± SE (*n* = 3). (f) Transcriptional abundances of circadian rhythm‐related genes in the third leaf of plants in the mutant and overexpression lines at stage V6 (34 DAG), grown under LD conditions. Data are means ± SE (*n* = 3). (See also Figure S7).

To elucidate the roles of ZmCOP1a and ZmCOP1b in regulating maize flowering, we assessed flowering‐related traits under LD and SD conditions. Under both photoperiods, the *zmcop1a* mutant flowered 2.8–3.0 days earlier and silked 2.0–1.5 days earlier than WT plants, whereas the *ZmCOP1a‐OE* lines flowered approximately 3.0–3.5 days later. Although the *zmcop1b* mutant did not exhibit significant changes in flowering or silking time compared with B73, the *ZmCOP1b‐OE* lines flowered 3.5 days later and silked 4.0–5.5 days later (Figure 3a,b,d). Additionally, the *zmcop1a/b* double mutant displayed severely retarded growth and developmental defects in both tassel and ear formation under field conditions (Figures 2g,h and 3c). Molecular analysis revealed upregulation of florigen genes (*ZCN7*, *ZCN8* and *ZCN12*) and flowering markers (*ZMM3*, *ZMM4* and *ZMM15*) in the *zmcop1a* mutant; their expression was strongly suppressed in the overexpression lines. The opposite expression pattern was observed for key photoperiod repressor genes (*ZmPRR37a*, *ZmPRR37b*, *ZmPRR95a*, *ZmPRR95b*, *ZmCCA1* and *ZmCCT9*) (Figure 3e,f). These results demonstrate that ZmCOP1a and ZmCOP1b function as repressors of flowering in maize.

### 2.4 ZmCOP1a and ZmCOP1b Regulate Flowering Time Through Interaction With ZmCOL3

**FIGURE 4:**
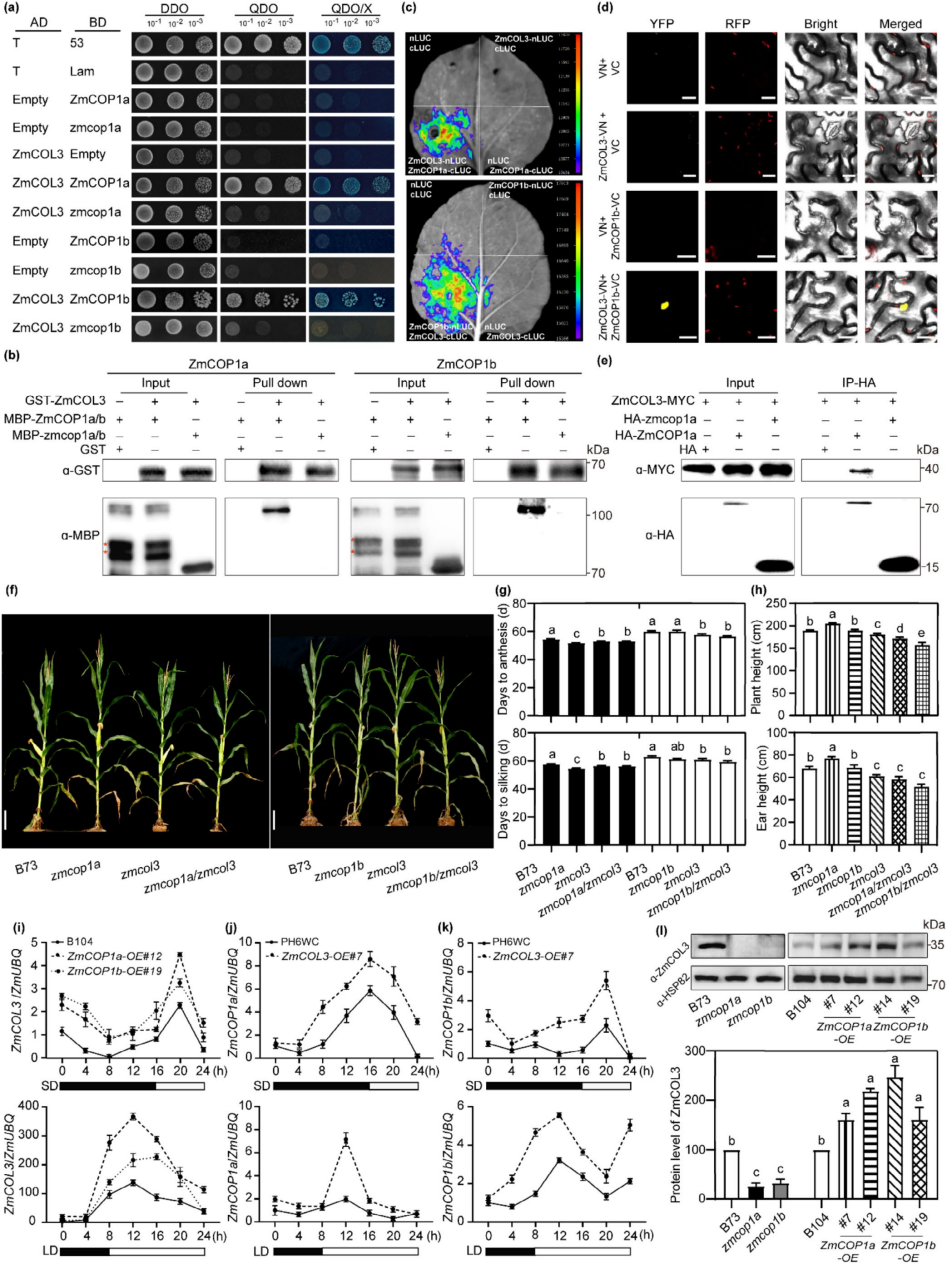
Both ZmCOP1a and ZmCOP1b regulate flowering time by interacting with ZmCOL3. (a) Yeast two‐hybrid (Y2H) assay showing physical interactions of ZmCOP1a and ZmCOP1b with ZmCOL3. (b) Pull‐down assay confirming in vitro interactions of ZmCOP1a and ZmCOP1b with ZmCOL3. Asterisks indicate non‐specific bands. (c) Luciferase complementation imaging assay validating interactions of ZmCOP1a and ZmCOP1b with ZmCOL3 in tobacco leaves. (d) Bimolecular fluorescence complementation assay confirming the interaction between ZmCOP1b and ZmCOL3 in tobacco leaves. Scale bar = 20 μm. (e) Co‐immunoprecipitation assay verifying the interaction between ZmCOP1a and ZmCOL3. (f) Field phenotypes of B73 (WT), *zmcop1a*, *zmcop1b*, *zmcol3*, *zmcop1a/zmcol3* and *zmcop1b*/*zmcol3* at the flowering stage. Scale bar = 20 cm. (g, h) Statistical analysis of flowering time, silking time, plant height and ear height corresponding to (f). (*n* ≥ 15). (i–k) Transcriptional abundances of *ZmCOL3* in *ZmCOP1a* and *ZmCOP1b* transgenic lines (i), or *ZmCOP1a* (j) and *ZmCOP1b* (k) in the *ZmCOL3* transgenic line under LD and SD conditions. (l) ZmCOL3 protein levels in single mutant and overexpression lines of *ZmCOP1a* and *ZmCOP1b*, with HSP82 as an internal reference. Data are means ± SE (*n* = 3). (See also Figures S8–S12).

To elucidate the molecular mechanism by which ZmCOP1a and ZmCOP1b regulate flowering time, we performed a yeast two‐hybrid (Y2H) assay to screen for interacting proteins. Among the candidates identified was the C2C2 CO‐like transcription factor ZmCOL3 (*Zm00001d017176*). Both ZmCOP1a and ZmCOP1b were found to interact with ZmCOL3 in their full‐length forms. However, when specific mutations were introduced into ZmCOP1a or ZmCOP1b, interaction with ZmCOL3 was abolished, as confirmed through Y2H assays (Figure 4a; Figure S8). This interaction was further validated by multiple independent approaches, including pull‐down, firefly luciferase complementation imaging, bimolecular fluorescence complementation (BiFC) and co‐immunoprecipitation (Co‐IP) assays (Figure 4a–e). Notably, truncated versions of ZmCOP1a and ZmCOP1b lacking either the RING domain (ZmCOP1‐ΔR) or the coiled‐coil domain (ZmCOP1‐ΔCC) still interacted with ZmCOL3 (Figure S8). These results indicate that the C‐terminal WD40 domain of ZmCOP1s is essential for interaction with ZmCOL3, although the presence of either the RING or coiled‐coil domain is also required for a functional binding interface.

To investigate the role of ZmCOL3 in flowering regulation, a *zmcol3* mutant was identified from the Maize EMS‐induced Mutant Database (MEMD; http://maizeems.qlnu.edu.cn/) (Lu et al. 2018). This mutant carries a G‐to‐A substitution at position 204 in the CDS, resulting in a premature stop codon (Trp68‐to‐stop) and reduced protein accumulation. Under LD conditions, the *zmcol3* mutant flowered 1.5 days earlier and silked 1.2 days earlier than WT plants, accompanied by significant reductions in plant height and ear height (Figure S9a,c–f). In contrast, *ZmCOL3* overexpression lines (*ZmCOL3‐OE* line #1 and *#7*, corresponding to transgenic lines 26–12 and 1–4 described in Jin et al. 2018) exhibited delayed flowering and silking (Figure S8b,c–f). These phenotypic changes were consistent with altered expression of florigen genes (*ZCN7*, *ZCN8* and *ZCN12*) and flowering markers (*ZMM3*, *ZMM4* and *ZMM15*) (Figure S9g,h). Together, these results support a model in which ZmCOP1a and ZmCOP1b interact with ZmCOL3 to delay flowering in maize, likely through repression of the florigen pathway.

To determine the genetic relationship between *ZmCOP1a*/*ZmCOP1b* and *ZmCOL3*, we generated the double mutants *zmcop1a*/*zmcol3* and *zmcop1b*/*zmcol3*. These double mutants exhibited flowering time, silking time, plant height and ear height phenotypes similar to those of the *zmcol3* single mutant (Figure 4f–h). Under both LD and SD conditions, *ZmCOP1a*/*ZmCOP1b* and *ZmCOL3* exhibited mutually enhanced transcription, with peak expression observed 4 h after W exposure. An exception was noted for *ZmCOP1a* expression in the *ZmCOL3‐OE* background under SD conditions, which did not lead to significant upregulation (Figure 4i–l). Compared with the B73 WT line, both transcript and protein levels of *ZmCOL3* were reduced in the *zmcop1a* and *zmcop1b* mutants. In contrast, the *ZmCOP1a‐OE* and *ZmCOP1b‐OE* lines (in the B104 background) showed substantially elevated *ZmCOL3* expression and accumulation (Figure 4i and Figure S10a). However, the expression of *ZmCOP1a* and *ZmCOP1b* remained unaltered in the *zmcol3* mutant (Figure S10b). These results indicate that *ZmCOP1a* and *ZmCOP1b* positively regulate *ZmCOL3* at both the transcriptional and protein levels, and that all three genes function within a common pathway to repress flowering in maize, with *ZmCOL3* acting epistatically (Figure S11).

To investigate whether ZmCOL3 stability is regulated through the 26S proteasome pathway, we treated 10‐day‐old B73 seedlings with the proteasome inhibitor MG132 (7 μM, 14 h) under dark and W light conditions. Immunoblot analysis revealed that ZmCOL3 accumulation was higher in dark‐grown seedlings compared to light‐grown seedlings without MG132 treatment, consistent with light‐dependent destabilisation. Strikingly, MG132 treatment abolished the difference between dark and W conditions, and importantly, did not further increase ZmCOL3 protein levels in dark‐grown seedlings compared to the untreated control (Figure S12). These results demonstrate that ZmCOL3 is not degraded via the 26S proteasome pathway, suggesting that ZmCOP1s stabilise ZmCOL3 through a non‐proteolytic mechanism.

### 2.5 *ZmCOP1s* and *ZmCOL3* Coregulate Flowering Time and Yield‐Related Genes

To assess the impacts of *ZmCOP1a*, *ZmCOP1b* and *ZmCOL3* on global gene expression, we analysed transcriptome data from *ZmCOL3‐OE* lines (NCBI Bioproject PRJNA403977) (Jin et al. 2018), as well as the *zmcop1a* and *zmcop1b* mutants. Raw sequencing data from the B73 and mutant lines were of high quality (Q30 > 95.26%). In total, 423.58 million clean reads were obtained, of which more than 97.71% were successfully mapped to the B73 RefGen_v4 reference genome (http://ftp.ensemblgenomes.org/pub/plants/release‐48/fasta/zea_mays/dna/) (Table S1). Correlation analysis of fragments per kilobase of transcript per million mapped reads (FPKM) values across biological replicates indicated high reproducibility, with correlation coefficients (R) ranging from 0.94 to 0.99 (Figure S13a). Principal component analysis (PCA) clearly separated the samples by genotype, with the two principal components (PC1 and PC2) explaining 63.88% and 22.15% of the total variance, respectively (Figure S13b).

**FIGURE 5:**
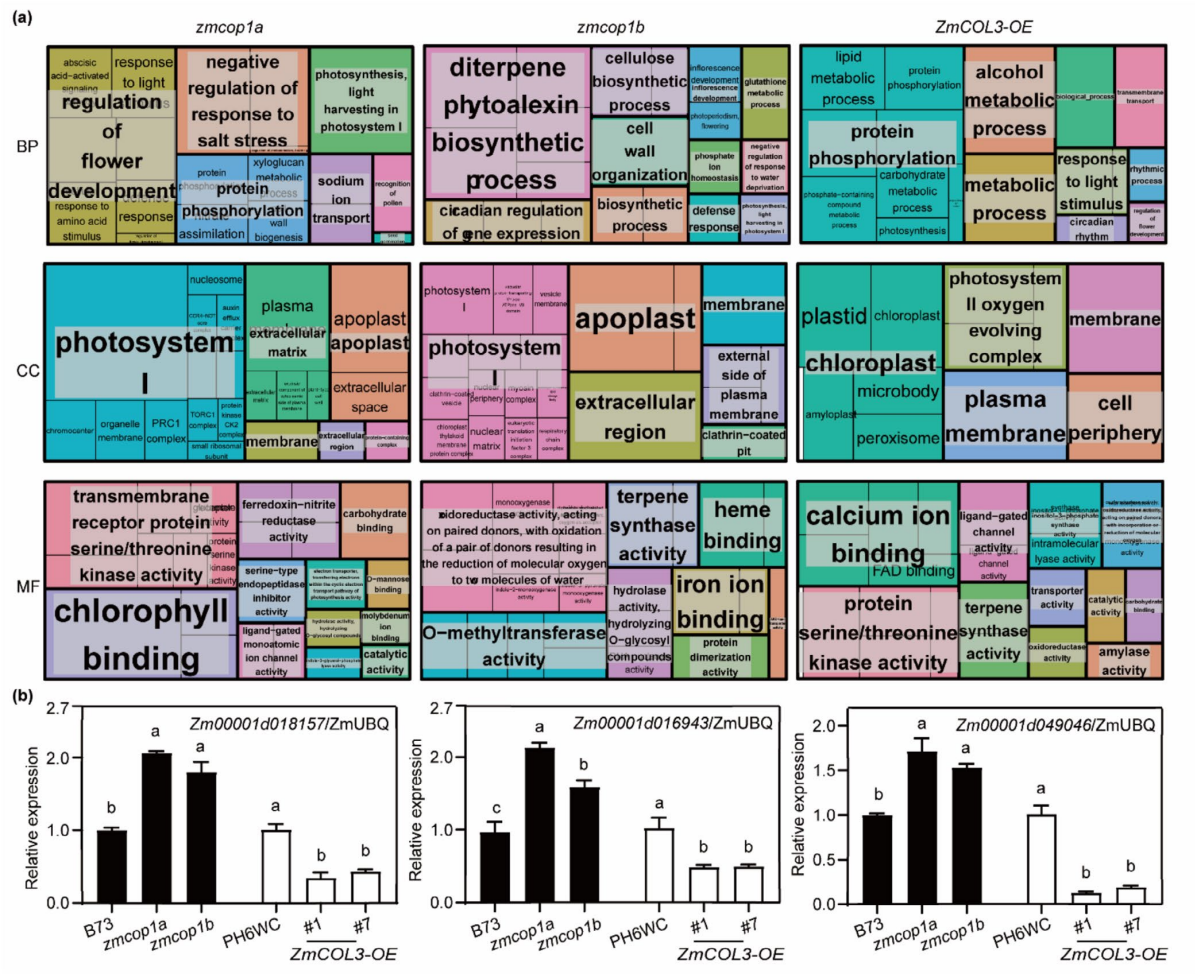
Transcriptomic analysis identifies photosynthesis as a major downstream pathway of the ZmCOP1–ZmCOL3 module. (a) Gene Ontology enrichment analysis of differentially expressed genes (DEGs) enriched in biological processes (BP), cellular components (CC) and molecular functions (MF). Treemap visualisation was generated using REVIGO, with rectangle sizes representing the significance level, based on thresholds of |log_2_(fold change)| > 2 and false discovery rate (FDR) < 0.05. (b) Transcript abundances of photosynthesis‐related DEGs (*Zm00001d018157*, *Zm00001d016943* and *Zm00001d049046*). Different letters indicate significant differences (*p* < 0.05, ANOVA followed by Turkey's test). (See also Figure S13).

Compared with the B73 WT line, the *zmcop1a*, *zmcop1b* and *ZmCOL3‐OE* genotypes exhibited distinct transcriptomic changes: 264, 257 and 1888 genes were upregulated, and 625, 279 and 1716 genes were downregulated, respectively (Figure S13c and Table S2). Gene Ontology (GO) and Kyoto Encyclopedia of Genes and Genomes (KEGG) analyses revealed significant enrichment of differentially expressed genes (DEGs) in biological processes and pathways associated with circadian rhythm, floral development, chloroplast organisation, photosystem assembly and photosynthesis (Figure 5a; Tables S2–S4). Key photosynthesis‐related genes [*Zm00001d018157* (Li et al. 2025; Shen et al. 2022), *Zm00001d016943* (Shen et al. 2022) and *Zm00001d049046* (Lundquist et al. 2013)] were upregulated in the loss‐of‐function mutants but downregulated in the *ZmCOL3* overexpression lines (Figure 5b; Table S2). These findings suggest that *ZmCOP1a*, *ZmCOP1b* and *ZmCOL3* converge to modulate flowering time and yield‐related traits through the transcriptional regulation of circadian rhythm and photosynthetic pathways.

### 2.6 The ZmCOP1s–ZmCOL3 Module Regulates Kernel Size and Yield in Maize

**FIGURE 6:**
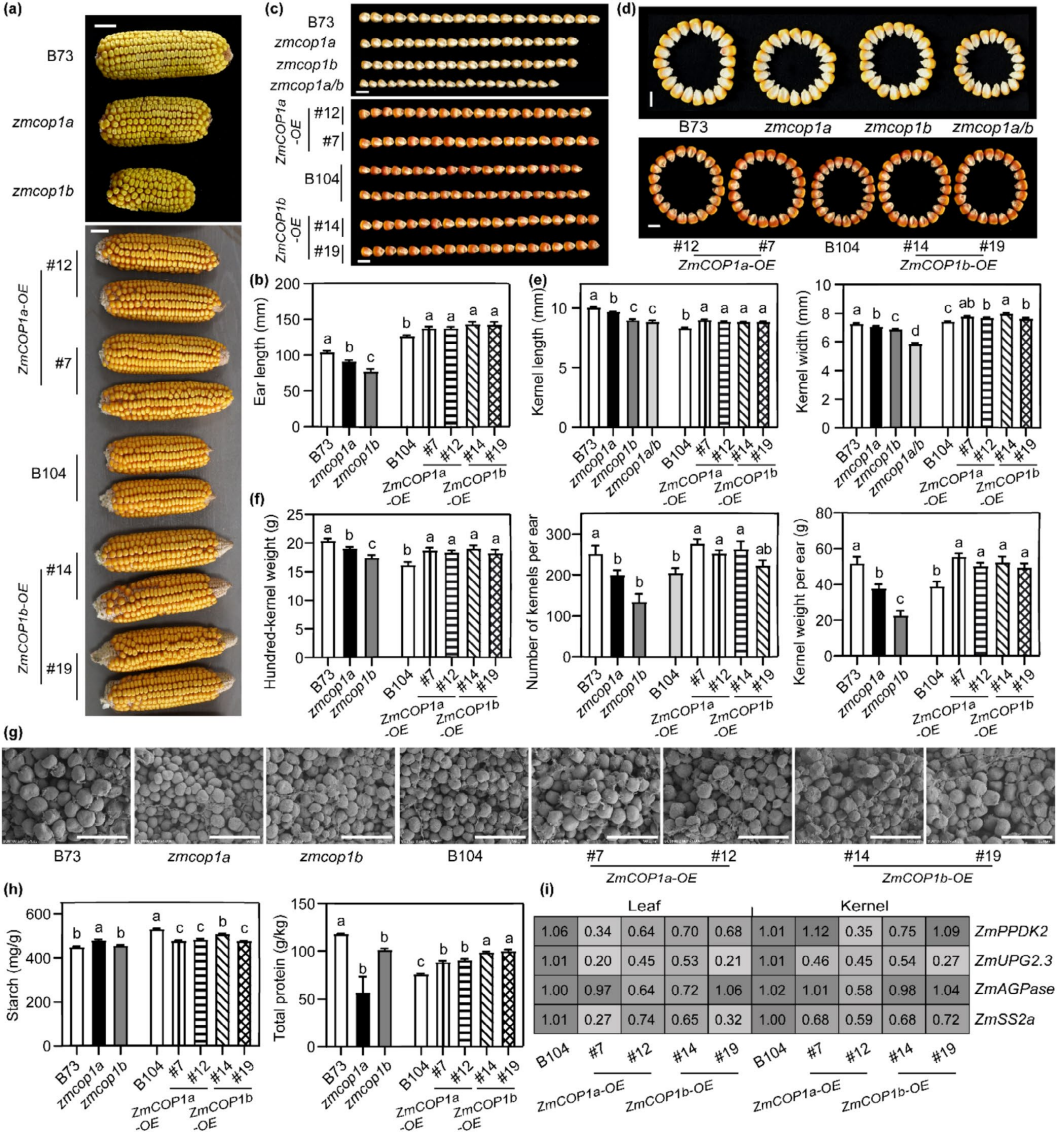
Both ZmCOP1a and ZmCOP1b modulate kernel size, starch–protein balance and yield. (a, b) Ear morphology of WT1, WT2, single mutant, double mutant and overexpression lines of *ZmCOP1a* and *ZmCOP1b grown under* SD conditions for 87 days. Scale bar = 2 cm. *n* ≥ 10. (c–e) Kernel length and width of indicated genotypes corresponding to (a) (*n* ≥ 50). Scale bar = 1 cm. (f) Hundred‐kernel weight, kernel number per ear and kernel weight per ear of indicated genotypes corresponding to (a) (*n* ≥ 10). (g) Scanning electron micrographs of mature kernel endosperm of indicated genotypes corresponding to (a). Scale bar = 50 μm. (h) Starch and total protein content in mature kernels of indicated genotypes corresponding to (a). Data are presented as means ± SE (*n* = 3). (i) Quantitative reverse‐transcription polymerase chain reaction expression analysis of starch metabolic genes in the third leaf of plants at the V6 stage and 15‐DAP kernels of WT2 and overexpression lines of *ZmCOP1a* and *ZmCOP1b*. Data are means ± SE (*n* = 3). (See also Figures S14–S16).

To quantify assess the impact of the ZmCOP1s–ZmCOL3 module on grain yield, we performed comprehensive phenotyping of ear and kernel‐related traits. Compared with the B73 WT line, the loss‐of‐function mutants *zmcop1a*, *zmcop1b*, *zmcol3*, *zmcop1a*/*zmcol3* and *zmcop1b*/*zmcol3* double mutants exhibited reductions in ear length (13.80%, 26.07%, 16.08%, 29.32% and 27.91%), kernel number per ear (20.94%, 46.63%, 33.07%, 18.96% and 20.00%), kernel weight per ear (24.29%, 56.14%, 37.14%, 42.10% and 34.31%), kernel length (3.77%, 10.81%, 12.20%, 9.51% and 5.37%), kernel width (2.75%, 5.635%, 7.03%, 7.65% and 14.97%), and hundred‐kernel weight (6.32%, 14.41%, 16.08%, 17.35% and 11.13%), respectively. The *zmcop1a/b* double mutant showed the most severe defects, suggesting partial functional redundancy between ZmCOP1a and ZmCOP1b. Notably, no significant differences were observed in kernel number per ear or kernel weight per ear between the single and double mutants, suggesting that *ZmCOP1s* and *ZmCOL3* function in the same genetic pathway regulating yield traits, with limited additive effects in double mutant combinations. Conversely, overexpression lines of *ZmCOP1s* (2.96%–41.74%) and *ZmCOL3* (10.18%–44.12%) showed increases in the same traits (Figure 6a–f; Figures S14a–f and S15; Table S5). Scanning electron microscopy further revealed that kernels from the mutants accumulated numerous small, densely packed starch granules, while overexpression lines produced fewer but significantly larger granules (Figure 6g; Figure S14h).

Compositional analysis revealed that the starch content of the *ZmCOP1a/b‐OE* lines was 89.52%–95.64% that of the WT lines, whereas total protein content was 1.2–1.3‐fold higher in the overexpression lines. Conversely, the corresponding mutants showed a modest increase in starch and a pronounced reduction in total protein, which fell to 47.93%–85.56% of WT levels (Figure 6h). In the *ZmCOL3‐OE#7* line, both starch and total protein decreased by less than 10% compared with the control (96.09% and 90.94%, respectively), these components were 1.05‐ and 1.23‐fold higher in the *zmcol3* mutant, respectively (Figure S14g). GO enrichment and quantitative reverse transcription–polymerase chain reaction (qRT‐PCR) analyses confirmed that DEGs were strongly enriched in starch and carbohydrate metabolic pathways (Figure 5a; Tables S3 and S4). Consistent with these findings, the *ZmCOP1a*/*b‐OE* and *ZmCOL3‐OE* lines downregulated key starch synthesis genes such as *ZmPPDK2*, *ZmSSIIa*, *ZmUPG2.3* and *ZmAGPase* (Zhan et al. 2024; Chen et al. 2023, 2025; Wang et al. 2022) (Figure 6i; Figure S14i). Furthermore, nine zein genes were upregulated in the *ZmCOP1a/b‐OE* kernels—consistent with the high‐protein phenotype—whereas they were broadly downregulated in the *ZmCOL3‐OE* lines (Figure S16). These results demonstrate that the ZmCOP1s–ZmCOL3 module reprograms carbon and nitrogen allocation by repressing starch biosynthesis and enhancing storage protein accumulation, leading to increased kernel size and synchronised yield improvement.

## 3 Discussion

### 3.1 Conserved Yet Divergent Roles of ZmCOP1a and ZmCOP1b

The *Arabidopsis cop1‐4* null mutant exhibits constitutive photomorphogenesis and early flowering (Deng et al. 1992; Ordoñez‐Herrera et al. 2018; Zhou and Deng 2025). Both maize paralogs, *ZmCOP1a* and *ZmCOP1b*, retain the conserved RING–coiled‐coil–WD40 domain architecture and fully complement these *Arabidopsis* mutant phenotypes. Specifically, *ZmCOP1a*/*cop1‐4* and *ZmCOP1b*/*cop1‐4* transgenic lines restore elongated hypocotyls (Figure 1a; Figure S3a–d) and delay flowering (Figure S7), confirming that the core COP1 function is conserved across eudicots and monocots. Despite such functional conservation, the two paralogs have undergone subfunctionalization. *ZmCOP1a* expression was considerably enriched in photosynthetic leaves and strongly induced by light, whereas *ZmCOP1b* was ubiquitously expressed at substantially lower levels (Figure 1b–d). Consistent with this divergence, the *zmcop1a* mutant flowered 2.8 days earlier under LD conditions and 2.0 days earlier under SD conditions (Figure 3a,d); the *zmcop1b* mutants showed no significant change in flowering time, indicating that ZmCOP1a acts as the dominant repressor of flowering in maize. Overexpression of either paralog reduced anthocyanin and increased chlorophyll accumulation (Figure 2; Figure S4), consistent with previous reports in dicots (Li et al. 2012; Maier and Hoecker 2015), although these effects were consistently stronger for ZmCOP1a. At the subcellular level, both ZmCOP1a and ZmCOP1b localised to the nucleus and cytoplasm, but ZmCOP1a exhibited significantly stronger nuclear signal intensity (Figure 1e; Figure S1), suggesting the presence of monocot‐specific interactors that may fine‐tune the activity and partitioning of each paralog.

### 3.2 The ZmCOP1s–ZmCOL3 Module Cooperatively Represses Maize Flowering

The photoperiod pathway integrates circadian signals to precisely control flowering time in maize (Yang et al. 2024). In this pathway, the evening complex, which comprises *ZmELF3.1*/*3.2*, *ZmELF4.1*/*4.2* and *ZmLUX1*/*2*—suppresses the expression of flowering repressors such as *ZmPRR37*/*73*, *ZmCCT9*/*10* and *ZmCOL3*, thereby de‐repressing florigen genes *ZCN7*/*8*/*12* and promoting flowering (Huang et al. 2018; Jin et al. 2018; Zhao et al. 2023). Interestingly, the CCT‐domain transcription factor ZmCOL3 upregulates *ZmCCT* genes and delays flowering (Jin et al. 2018; Su et al. 2021), suggesting that its repressive function is modulated by upstream regulators. We demonstrated that ZmCOP1a and ZmCOP1b physically interacted with ZmCOL3 (Figure 4a–e; Figure S8). This interaction produced two synergistic regulatory effects: transcriptionally, ZmCOP1a/b and ZmCOL3 exhibited mutually enhanced expression, and post‐translationally, ZmCOP1a/1b stabilised the ZmCOL3 protein in a manner independent of the 26S proteasome pathway (Figure 4i–l; Figure S10).

The molecular basis of ZmCOP1‐mediated ZmCOL3 stabilisation represents a paradigm shift from the canonical COP1 function. Based on immunoblotting analysis (Figure 4i; Figure S12) and recent advances in COP1‐mediated non‐proteolytic ubiquitination (Liu et al. 2025), we propose that ZmCOP1s stabilise ZmCOL3 through K63‐linked polyubiquitination, which functionally inactivates ZmCOL3 without promoting its degradation. This model is supported by three lines of evidence: (1) ZmCOP1s interact with and co‐localise with ZmCOL3 in the nuclear speckles (Figure 1e; Jin et al. 2018); (2) the epistatic relationship between *zmcop1* and *zmcol3* single and double mutants suggests they function in the same pathway with limited additive effects (Figure S15); and (3) MG132 treatment experiments demonstrate that ZmCOL3 accumulation is insensitive to 26S proteasome inhibition (Figure S12), consistent with K63‐linked rather than K48‐linked ubiquitination (Liu et al. 2025). We hypothesize that this “stable but inactive” state allows rapid activation of ZmCOL3 upon light‐induced inhibition of ZmCOP1s, providing an efficient regulatory switch for flowering time control. Alternatively, ZmCOP1 binding may protect ZmCOL3 from other E3 ligases through steric hindrance or recruitment of deubiquitinating enzymes.

This “stabilise‐and‐activate” mechanism parallels the darkness‐dependent accumulation of PIF3, shade‐induced enrichment of PIF5 (Sharma et al. 2023), and ultraviolet B‐mediated stabilisation of HY5 (Ren et al. 2019), highlighting the conserved role of COP1 as a central protein stabiliser within diverse light‐signalling networks. However, distinct from these examples, ZmCOP1s appear to maintain ZmCOL3 in a functionally inactive state through non‐proteolytic ubiquitination, representing a nuanced adaptation of the COP1 regulatory repertoire.

Upon stabilisation by ZmCOP1a/1b, ZmCOL3 upregulated key circadian and floral repressors including *ZmPRR37a*/*b*, *ZmPRR95a*/*b*, *ZmCCA1* and *ZmCCT9*, which then suppressed the expression of florigen genes *ZCN7*/*8*/*12* and floral meristem identity genes *ZMM3*/*4*/*15*, ultimately delaying flowering (Figure S11). Convergent regulatory architecture has been identified in both rice (through the OsphyB–OsCOL4–Ehd1 module) (Lee et al. 2010) and soybean (via GmCOL3a–E1–FT2a/5a) (Gao et al. 2025), suggesting a conserved, precise mechanism for photoperiodic flowering control across divergent crop species.

### 3.3 Delayed Flowering Redirects Carbon–Nitrogen Allocation to Enhance Yield and Nutritional Quality

Flowering time is a critical determinant of maize yield stability. Developmental asynchrony between the tassel and ear can lead to kernel abortion (Li, Liu, et al. 2023), whereas mistimed flowering results in inefficient photosynthate allocation and substantial yield loss (Niu et al. 2024). Our integrated phenotypic, compositional and transcriptomic analyses demonstrated that the ZmCOP1s–ZmCOL3 module delayed flowering under both LD and SD conditions and significantly increased kernel weight per ear (Figure 6; Figure S14). In the *ZmCOP1a/b‐OE* lines, carbon–nitrogen metabolism was reprogrammed during grain filling, leading to broad downregulation of starch biosynthesis genes and strong enhancement of zein synthesis, which represents over 60% of endosperm protein (Figure 6; Figure S16). This shift aligns with the established starch–protein trade‐off model (Chen et al. 2025; Zhang, Wei, et al. 2025). In contrast, *ZmCOL3* overexpression simultaneously suppressed both starch and protein biosynthesis (Figures S14 and S16), challenging the presumed negative correlation (Chen et al. 2023; Li et al. 2024; Yang et al. 2021). The mechanistic basis for this distinct regulatory outcome requires further investigation.

Collectively, these findings position the ZmCOP1s–ZmCOL3 module as a central regulator that simultaneously enhances yield and nutritional quality through a coordinated strategy of delayed flowering and carbon–nitrogen reallocation. This module provides a promising target for genome editing or allele stacking aimed at developing high‐yield, nutritionally improved and climate‐resilient maize varieties.

## 4 Conclusions

We have systematically identified and functionally characterised two maize orthologs of *Arabidopsis COP1*—*ZmCOP1a* and *ZmCOP1b*. These proteins conserved their roles in photomorphogenic signalling across species and physically interacted with the transcription factor ZmCOL3 to enhance its protein stability, leading to suppression of key flowering integrators, including *ZCN7*/*8*/*12* and *ZMM3*/*4*/*15*. The resulting delay in flowering coincided with significant increases in kernel size and hundred‐kernel weight, as well as a reconfigured kernel nutrient composition. Therefore, the ZmCOP1s–ZmCOL3 module coordinately regulates the timing of flowering and core components of yield quantity and quality, offering both mechanistic insight and practical genetic targets for breeding high‐yield, nutritionally improved maize.

## 5 Materials and Methods

### 5.1 *Arabidopsis* Growth Conditions

The *cop1‐4* (Yang et al. 2005), *ZmCOP1a*/*cop1‐4* and *ZmCOP1b*/*cop1‐4* lines were generated in the *Arabidopsis thaliana* ‘Col‐0’ ecotype background. *Arabidopsis* seeds were surface‐sterilised and sown as previously described (Chen, Fan, et al. 2024). Seedlings were cultivated at 22°C for 5 days under Dk or W (17 μmol·m^−2^·s^−1^). Hypocotyl length was measured in more than 50 seedlings; anthocyanin and chlorophyll contents were quantified using three biological replicates, each consisting of 100 seedlings. Relative expression levels of the *CAB* and *CHS* genes were also analysed in three independent biological samples. For flowering time, plant height and rosette leaf number assays, plants were grown under LD (16‐h light/8‐h dark) or SD (8‐h light/16‐h dark) conditions. To analyse the expression of flowering‐related genes (*FT*, *CO* and *SOC1*), whole seedlings were harvested at 10 days under LD conditions and at 30 days under SD conditions, with three biological replicates per condition.

### 5.2 Maize Growth Conditions

Maize plants of the inbred B73 line were grown in Zhengzhou, Henan Province, for 60 days. Samples from tassel, ear, silk, leaf, sheath, stem and root tissues were collected to analyse tissue‐specific expression patterns of *ZmCOP1a* and *ZmCOP1b*. To assess the circadian expression of *ZmCOP1a*, *ZmCOP1b* and *ZmCOL3*, seedlings were cultivated under LD or SD conditions for 10 days; then, leaf samples were harvested for qRT‐PCR, with three biological replicates.

For phenotypic measurements under different light conditions, maize seeds were sown in soil and grown for 10 days at 26°C under the following conditions: Dk and W, 70 μmol·m^−2^·s^−1^; FR, 2.5 μmol·m^−2^·s^−1^; R, 26 μmol·m^−2^·s^−1^; and B, 26 μmol·m^−2^·s^−1^. Illumination was supplied by a light‐emitting diode source (model F‐700; Hipoint, Kaohsiung, Taiwan, China), and light fluence rates were quantified using an HR‐350 spectrometer (Hipoint). First‐leaf sheath length, mesocotyl length, anthocyanin and chlorophyll contents and expression levels of *ZmCAB* and *ZmCHS* were determined in these seedlings.

Field trials for agronomic trait evaluation were conducted in Ledong, Hainan Province (18° N, 116° E; SD condition in winter) and Zhengzhou, Henan Province (34.8° N, 113.6° E; LD conditions in summer). Plants from the mutant, transgenic and control inbred lines were arranged in a randomised design. The measured traits included days to anthesis, days to silking, plant height, ear height and kernel‐related characteristics such as kernel length, width, hundred‐kernel weight, number per ear, weight per ear and starch and protein content (Zhang, Wei, et al. 2025).

### 5.3 Phylogenetic Analysis and Conserved Motif Identification of COP1 Homologues

Protein sequences of COP1 homologues from *Zea mays* (ZmCOP1a and ZmCOP1b), *Sorghum bicolor* (SbCOP1), *Oryza sativa* (OsCOP1), *Setaria italica* (SiCOP1a and SiCOP1b), *Triticum aestivum* (TaCOP1a, TaCOP1b and TaCOP1c), *Glycine max* (GmCOP1a and GmCOP1b) and *Arabidopsis* (AtCOP1) were obtained from the Ensembl Plants database (https://plants.ensembl.org/index.html). Multiple sequence alignment was performed using MEGA7 (Kumar et al. 2016). Phylogenetic reconstruction was performed and visualised using the neighbour‐joining method in MEGA7 (Kumar et al. 2016), supported by 1000 bootstrap replicates. Conserved motifs in COP1 protein sequences were predicted using the MEME suite (Bailey et al. 2009).

### 5.4 cDNA and qRT‐PCR

Total RNA was extracted from various tissues using TRNzol Universal Reagent (Tiangen, Beijing, China). Two micrograms of total RNA were reverse‐transcribed into cDNA using PrimeScript II reverse transcriptase (TaKaRa, Tokyo, Japan) with an oligo(dT)_18_ primer. The synthesised cDNA was diluted five‐fold and used as template for qRT‐PCR, which was conducted as previously described (Zhan et al. 2024). *ZmUBQ* (*Zm00001d015327*) and *AtACT2* (*AT3G18780*) were used as internal reference genes for maize and *Arabidopsis*, respectively (TableS6). The relative expression levels of target genes were calculated using the 2^−ΔΔCT^ method.

### 5.5 Subcellular Localization

Transient transformation was performed to determine the subcellular localization of ZmCOP1a and ZmCOP1b in both maize protoplasts and tobacco leaf epidermal cells. The CDSs of *ZmCOP1a*, *ZmCOP1b*, *ZmCOP1b‐ΔR* and *ZmCOP1b‐ΔCC* were cloned into the *pSuper1300* vector using *Hind*III and *Kpn*I restriction sites, downstream of the superpromoter, to generate the following C‐terminal GFP fusion constructs: ZmCOP1a–GFP, ZmCOP1b–GFP, ZmCOP1b‐ΔR–GFP and ZmCOP1b‐ΔCC–GFP. Maize protoplasts were isolated from etiolated seedlings of the B73 inbred line grown in Dk conditions for 2 weeks, as previously described (Zhang, Li, et al. 2025). For each transfection, approximately 10 μg of plasmid (including fusion constructs and the empty pSuper1300–GFP vector) was co‐transfected with a nuclear mCherry marker into 200 μL of protoplasts. After incubation in the dark at 28°C for 12 h, fluorescence signals were captured using a confocal microscope (FV1000; Olympus, Tokyo, Japan).

For localization in *Nicotiana benthamiana*, each GFP fusion construct was introduced into *Agrobacterium tumefaciens* strain *GV3101*. The bacterial suspensions were mixed with a strain containing the P19 silencing suppressor and a nuclear mCherry marker in an infiltration buffer composed of 150 μM acetosyringone, 10 mM MES (2‐(N‐morpholino) ethanesulfonic acid) and 10 mM MgCl_2_. After incubation in Dk conditions at 28°C for 2 h, the mixtures were infiltrated into the abaxial side of young *N. benthamiana* leaves. Plants were maintained under Dk conditions at 25°C for 3 days, then subjected to fluorescence imaging using a confocal microscope (FV1000; Olympus).

### 5.6 Identification of Maize EMS Mutants

The EMS‐induced mutants *zmcop1a* (EMS4‐080b8d), *zmcop1b* (EMS4‐11965c) and *zmcol3* (EMS 4‐07c8f3) were acquired from MEMD (http://maizeems.qlnu.edu.cn/) (Lu et al. 2018). To minimise genetic background noise, each mutant was backcrossed twice with the B73 inbred line as the recurrent parent. Homozygous mutants were selected from the resulting BC_2_F_3_ population and used for subsequent experiments. The *zmcop1a* and *zmcop1b* homozygous single mutants were crossed to generate F_1_ hybrids, which were then self‐pollinated to produce an F_2_ population. The *zmcop1a*/*b* double homozygous mutant was identified within the F_2_ generation by genotyping and sequencing.

### 5.7 Plasmid Construction and Plant Transformation

The CDSs of *ZmCOP1a* and *ZmCOP1b* were amplified by PCR using the gene‐specific primers ZmCOP1a/b‐F, ZmCOP1a‐R and ZmCOP1b‐R. The resulting PCR products were digested with *BamH*I and *Sac*I and ligated into the corresponding sites of the *pCAMBIA3301* vector, yielding the binary constructs *pCAMBIA3301‐ZmCOP1a‐FL* and *pCAMBIA3301‐ZmCOP1b‐FL*. All constructs were verified by sequencing. For maize transformation, the confirmed plasmids were introduced into *A. tumefaciens* strain *EHA105* via electroporation and subsequently transformed into maize inbred line B104 using an optimised *Agrobacterium*‐mediated method (Kang et al. 2022). For complementation in *Arabidopsis*, the same constructs were electroporated into *A. tumefaciens* strain *GV3101* and introduced into the *cop1‐4* mutant (Yang et al. 2005) via the floral dip method. Transgenic maize and *Arabidopsis* lines were selected based on Basta resistance and further validated by qRT‐PCR to confirm transgene expression. The primer sequences used in this study are listed in Table S6.

### 5.8 Anthocyanin and Chlorophyll Content Measurement

Anthocyanin and chlorophyll content levels were measured using established methods (Chen, Fan, et al. 2024; Zheng et al. 2013; Fankhauser and Casal 2004). For *Arabidopsis*, 300 seedlings per line were grown at 22°C for 5 days under Dk or W conditions. For maize, 0.5 g of seedlings were grown at 26°C for 10 days under Dk, W, FR R, or B conditions. Anthocyanin extraction and quantification were performed as follows: for *Arabidopsis*, relative anthocyanin content (ng/seedling) was calculated as (*A*_530_−0.25 × *A*₆₅₇)/100; for maize, relative anthocyanin content (mg/g) was calculated as (*A*_530_−0.25 × *A*_657_)/0.5. Chlorophyll content was determined as previously described (Fankhauser and Casal 2004). For *Arabidopsis*, chlorophyll concentration (μg/100 seedlings) was calculated as (18.71 × *A*_647_ + 7.15 × *A*_663_)/100. For maize, chlorophyll concentration (mg/g) was calculated as (18.71 × *A*_647_ + 7.15 × *A*_663_)/0.5. All measurements were performed with three biological replicates.

### 5.9 Y2H Assay

The CDS of *ZmCOL3* from maize inbred line B73 was cloned into the *pGADT7* vector (AD domain). The full‐length, mutated, truncated CDS fragments of *ZmCOP1a* and *ZmCOP1b* were individually inserted into the *pGBKT7* vector (BD domain). Recombinant AD and BD plasmids were co‐transformed into the Y2H Gold yeast strain. Transformants were selected and cultured on double dropout medium (−Leu/−Trp) and quadruple dropout medium (−Leu/−Trp/−His/−Ade) supplemented with X‐α‐Gal for 3 days. The pair AD‐T + BD‐53 was used as a positive control; AD‐T + BD‐Lam served as a negative control. Each interaction test was performed with three independent biological replicates, each containing three different concentration gradients. The primers used in this study are listed in TableS6.

### 5.10 Firefly Luciferase Complementation Imaging Assay

To examine interactions among ZmCOP1a, ZmCOP1b and ZmCOL3, we performed a luciferase complementation imaging assay in *N. benthamiana* leaves. Full‐length CDSs of *ZmCOP1a* and *ZmCOP1b* were cloned into the *pCAMBIA1300*–*cLUC* and *pCAMBIA1300*–*nLUC* vectors, respectively. The full‐length CDS of *ZmCOL3* was inserted into both *pCAMBIA1300*–*cLUC* and *pCAMBIA1300*–*nLUC*. Recombinant plasmids were introduced into *A. tumefaciens* strain *GV3101* and co‐infiltrated into leaves of 4‐week‐old *N. benthamiana* plants. After incubation in W condition light at 26°C for 48 h, a solution containing 0.5 mM luciferin was sprayed onto the leaves and they were incubated in DK condition for 10 min. Luminescence signals were captured using a low‐light cooled charge‐coupled device camera. The experiment included three biological replicates, each showing consistent results. Primer sequences used for vector construction are listed in TableS6.

### 5.11 Pull‐Down Assay

Full‐length CDSs of the genes of interest were cloned into the *pMAL‐c5x* (for maltose‐binding protein [MBP] tagging) or *pGEX4T‐1* (for glutathione S‐transferase [GST] tagging) vectors. MBP‐ and GST‐fused proteins were expressed in *Escherichia coli* strain Transetta *BL21* (TransGen Biotech, Beijing, China) by induction with 0.3 mM isopropyl β‐D‐1‐thiogalactopyranoside. Induction was performed for 4 h at 37°C, followed by 16 h at 4°C. Bacterial cultures were harvested and proteins purified according to established protocols (Jiang et al. 2025).

For the pull‐down assay, 10 μg of purified GST‐tagged protein was incubated with Pierce Glutathione Agarose (Thermo Fisher Scientific, Waltham, MA, USA) for 2 h at 4°C in 200 μL phosphate‐buffered saline (PBS) (10 mM Na_2_HPO_4_, 1.8 mM KH_2_PO_4_, 150 mM NaCl, 2.7 mM KCl, pH 7.3) supplemented with 0.1% Nonidet P‐40 and 1 mM phenylmethanesulfonyl fluoride (PMSF). After centrifugation at 500*g* for 5 min at 4°C, the supernatant was discarded. Then, 10 μg of MBP‐tagged protein and 200 μL of fresh PBS (with 0.1% Nonidet P‐40 and 1 mM PMSF) were added to the resin. The mixture was rotated at 4°C for 4 h and washed six times with 1.5 mL PBS containing 1% Triton X‐100 to remove non‐specifically bound proteins. Bound proteins were eluted and analysed by immunoblotting using anti‐MBP or anti‐GST antibodies (1:5000 dilution; Abmart, Berkeley Heights, NJ, USA).

### 5.12 Co‐IP Assay

The CDSs of the genes of interest were cloned into the *pAN580* vector (C‐terminal HA tag) or the *pSuper1300* vector (C‐terminal MYC tag). The recombinant plasmids *HA*–*ZmCOP1a* (or *HA*–*zmcop1a*) and *ZmCOL3*–*MYC* were co‐transfected into rice protoplasts. After 16 h of incubation, total protein was extracted using IP buffer (50 mM tris–HCl, pH 7.5, 150 mM NaCl, 0.2% Nonidet P‐40, 5 mM dithiothreitol, 1 mM PMSF) and pre‐cleared by incubation with pre‐washed Protein A/G beads for 30 min at 4°C. Following bead removal by centrifugation, an anti‐HA antibody was added to the supernatant, which was then incubated for 3 h at 4°C. Next, freshly washed Protein A/G beads were added and incubation continued for another 2 h. The beads were washed three times with 1× PBS to eliminate non‐specifically bound proteins. Bound proteins were eluted, denatured in loading buffer by boiling for 10 min, and analysed by immunoblotting using anti‐MYC and anti‐HA antibodies (1:5000 dilution; Abmart).

### 5.13 BiFC Assay

The CDSs of *ZmCOP1b* and *ZmCOL3* were cloned into the *pUC‐SPYNE* and *pUC‐SPYCE* vectors, respectively. The resulting constructs were introduced into *A. tumefaciens* strain *GV3101* and co‐infiltrated into *N. benthamiana* leaves. After infiltration, the plants were incubated for 48–72 h under normal growth conditions. Yellow fluorescent protein fluorescence was observed to detect protein–protein interaction under a confocal laser scanning microscope (LSM710, Zeiss, Oberkochen, Germany or A1+; Nikon, Tokyo, Japan).

### 5.14 Antibody Production Against ZmCOL3

A rabbit polyclonal antibody specific to ZmCOL3 was generated by HuaAn Biotechnology (Hangzhou, China). The peptide antigen (CPAQGEAVAEDYGSS) was synthesised and conjugated to keyhole limpet hemocyanin (KLH) as a carrier protein. The antibody was raised in rabbits immunised with the peptide and subsequently affinity‐purified from serum. For immunoblot analysis, the purified anti‐ZmCOL3 antibody was used at a 1:1000 dilution.

### 5.15 Data Processing for RNA Sequencing

Total RNA was extracted from maize inbred line B73 and mutants *zmcop1a* and *zmcop1b* (3 genotypes × 3 biological replicates) using the mirVana miRNA Isolation Kit (Ambion, Austin, TX, USA), in accordance with the manufacturer's instructions. RNA integrity was verified; cDNA libraries were prepared and sequenced as previously described (Zhan et al. 2023). Publicly available RNA sequencing data for *ZmCOL3* were downloaded from the National Center for Bioinformation Sequence Read Archive (BioProject no. PRJNA403977) (Jin et al. 2018).

Clean reads from all samples were aligned to the *Zea mays* B73 RefGen_v4 reference genome (Ensembl Plants release‐48) using HISAT2 (Kim et al. 2019). Gene expression quantification, Pearson correlation analysis and PCA were performed using established methods (Zhan et al. 2023). DEGs were identified using thresholds of |log_2_(fold change)| > 1 and false discovery rate (FDR) ≤ 0.05. GO enrichment analysis of DEGs was conducted using agriGO v2.0 with the singular enrichment analysis tool (Tian et al. 2017); results were summarised using REVIGO (Supek et al. 2011). Functional annotation and KEGG pathway mapping of DEGs were performed using the KEGG database (Kanehisa et al. 2008).

### 5.16 Statistical Analysis

Statistical analyses were performed using GraphPad Prism software. Comparisons between two groups were conducted using Student's *t*‐test; those among three or more groups were performed using one‐way analysis of variance (ANOVA), followed by Tukey's honest significant difference (HSD) test for multiple comparisons. Significance was evaluated using a threshold of *p* < 0.05.

## 6 Accession Numbers

Accession numbers Sequence data from this article can be found in the MaizeGDB data library under accession numbers: *AtCOP1* (*AT2G32950*); *ZmCOP1a* (*Zm00001d018207*); *ZmCOP1b* (*Zm00001d052138*); *ZmHY5* (*Zm00001d015743*); *ZmGA2ox10* (*Zm00001d012712*); *ZmCOL3* (*Zm00001d017176*); *ZmUBQ* (*Zm00001d015327*); *ZmCHS* (*Zm00001d007403*); *ZCN7* (*Zm00001d038725*); *ZCN8* (*Zm00001d010752*); *ZCN12* (*Zm00001d043461*); *ZmCCT1* (*Zm00001d024909*); *ZmPRR37a* (*Zm00001d022590*); *ZmPRR73* (*Zm00001d047761*); *ZmCCA1* (*Zm00001d049543*); *ZmPRR95a* (*Zm00001d021291*); *ZmPRR95b* (*Zm00001d006212*); *ZmCCT9* (*Zm00001d000176*); *ZMM15* (*Zm00001d013259*); *ZMM4* (*Zm00001d034045*); *ZMM3* (*Zm00001d045231*); *ZmPPDK2* (*Zm00001d010321*); *ZmUPG2.3* (*Zm00001d015008*); *ZmSS2a* (*Zm00001d002256*); *ZmAGPase* (*Zm00001d033910*); *19 kDa α‐zein B3* (*Zm00001eb303160*); *19 kDa α‐zein D2* (*Zm00001eb030160*); *19 kDa α‐zein D4* (*Zm00001d030855*); *22 kDa α‐zein8b* (*Zm00001d048809*); *24 kDa α‐zein8* (*Zm00001d048813*); *15 kDa β‐zein* (*Zm00001d035760*); *50 kDa γ‐zein* (*Zm00001d020591*); *10 kDa δ‐zein* (*Zm00001d045937*); *18 kDa δ‐zein* (*Zm00001d037436*).

## Author Contributions

J.Y., Y.Z. and X.L. designed the research and wrote the manuscript. J.T. contributed to manuscript revision. S.Y. and Y.Z. performed most of experiments and analysed the data. L.C. conducted the Y2H assay and generated the *ZmCOP1a/b* transgenic lines. W.Z. conducted the RNA‐seq. Y.S. and S.Z. were responsible for field planting and management. S.Y., Y.Z., K.Z., L.D., H.H., L.S., H. L. and S.W. participated in the identification and data statistics of field materials. X.L. contributed maize transgenic plants of *ZmCOL3‐OE*.

## Funding

This work was supported by the Science and Technology Project of Xizang Autonomous Region of China (XZ202401ZY0072, XZ202401ZY0103), the Science and Technology Plan Project of Lhasa City (LSKJ202551), the Lhasa Regional Science and Technology Collaborative Innovation Project in 2022 (QYXTZX‐LS2022‐01), the Science and Technology Plan Project of Tibet Plateau Seed Industry Breeding Technology Innovation Center (LSQSCNYQ2025007) and the National Natural Science Foundation of China (U23A20186).

## Conflicts of Interest

The authors declare no conflicts of interest.

## Supporting information


**Figure S1:** Subcellular localization of ZmCOP1a and ZmCOP1b in tobacco leaf epidermal cells.
**Figure S2:** Identification of *ZmCOP1a* and *ZmCOP1b* mutants and transgenic lines.
**Figure S3:** ZmCOP1a and ZmCOP1b promote seedling etiolation in *Arabidopsis*.
**Figure S4:** Phenotypes of inbred line B73 (wild type 1, WT1), inbred line B104 (transgenic background, WT2), single mutants, double mutant and overexpression lines of *ZmCOP1a* and *ZmCOP1b* under far‐red (FR), red (R), or blue (B) light conditions.
**Figure S5:** Both *ZmCOP1a* and *ZmCOP1b* transgenic lines reduce plant height and ear height in maize.
**Figure S6:** Molecular and biochemical evidences underlying ZmCOP1a/b‐mediated plant height reduction.
**Figure S7:** Both ZmCOP1a and ZmCOP1b delay flowering in *Arabidopsis*.
**Figure S8:** Interactions between different functional domains of ZmCOP1 with ZmCOL3 in yeast.
**Figure S9:** ZmCOL3 delays flowering under LD in maize.
**Figure S10:** *ZmCOP1a*/*ZmCOP1b* and *ZmCOL3* mutually enhance each other's transcription in maize.
**Figure S11:** Proposed regulatory module of ZmCOP1s–ZmCOL3 in controlling maize flowering time.
**Figure S12:** Effect of proteasome inhibitor MG132 on ZmCOL3 protein accumulation.
**Figure S13:** Differential gene expression in the *zmcop1a*, *zmcop1b* and *ZmCOL3‐OE* at the V6 stage.
**Figure S14:** ZmCOL3 modulates kernel development, starch‐protein composition and yield‐related traits.
**Figure S15:** Phenotypic analysis of yield‐related traits in *zmcop1a/zmcol3* and *zmcop1b/zmcol3* double mutants.
**Figure S16:** Expression analysis of zein genes in 15‐DAP kernels by RT‐qPCR.

**Table S1:** Statistics of the sequencing reads and alignment results in RNA‐seq.
**Table S2:** Differentially expressed genes (DEGs) in *zmcop1a*, *zmcop1b* and *ZmCOL3‐OE*.
**Table S3:** Gene ontology (GO) enrichment analyses of DEGs in *zmcop1a*, *zmcop1b* and *ZmCOL3‐OE*.
**Table S4:** Kyoto Encyclopedia of Genes and Genomes (KEGG) pathway analyses of DEGs in *zmcop1a*, *zmcop1b* and *ZmCOL3‐OE*.
**Table S5:** Comparison of grain size and yield characteristics.
**Table S6:** Primers used in this study.

## Data Availability

All data supporting the findings of this study are included in the manuscript and its Supporting Information, or from the corresponding author.
