# Supplementary material for: The ZmCOP1s–ZmCOL3 Module Enhances Late Flowering, Grain Yield and Grain Quality in Maize
Source: Plant Biotechnol J. 2026 Mar 18;24(6):4415–32. doi: 10.1111/pbi.70643 (PMC13205590; doi:10.1111/pbi.70643)
Supplement: Supplementary file 1 — Figure S1: Subcellular localization of ZmCOP1a and ZmCOP1b in tobacco leaf epidermal cells. Figure S2: Identification of ZmCOP1a and ZmCOP1b mutants and transgenic lines. Figure S3: ZmCOP1a and ZmCOP1b promote seedling etiolation in Arabidopsis. Figure S4: Phenotypes of inbred line B73 (wild type 1, WT1), inbred line B104 (transgenic background, WT2), single mutants, double mutant and overexpression lines of ZmCOP1a and ZmCOP1b under far‐red (FR), red (R), or blue (B) light conditions. Figure S5: Both ZmCOP1a and ZmCOP1b transgenic lines reduce plant height and ear height in maize. Figure S6: Molecular and biochemical evidences underlying ZmCOP1a/b‐mediated plant height reduction. Figure S7: Both ZmCOP1a and ZmCOP1b delay flowering in Arabidopsis. Figure S8: Interactions between different functional domains of ZmCOP1 with ZmCOL3 in yeast. Figure S9: ZmCOL3 delays flowering under LD in maize. Figure S10: ZmCOP1a/ZmCOP1b and ZmCOL3 mutually enhance each other's transcription in maize. Figure S11: Proposed regulatory module of ZmCOP1s–ZmCOL3 in controlling maize flowering time. Figure S12: Effect of proteasome inhibitor MG132 on ZmCOL3 protein accumulation. Figure S13: Differential gene expression in the zmcop1a, zmcop1b and ZmCOL3‐OE at the V6 stage. Figure S14: ZmCOL3 modulates kernel development, starch‐protein composition and yield‐related traits. Figure S15: Phenotypic analysis of yield‐related traits in zmcop1a/zmcol3 and zmcop1b/zmcol3 double mutants. Figure S16: Expression analysis of zein genes in 15‐DAP kernels by RT‐qPCR. [file PBI-24-4415-s002.docx]

**Supplemental Figures and Tables**

Figure S1 Subcellular localization of ZmCOP1a and ZmCOP1b in tobacco leaf epidermal cells.

Figure S2 Identification of *ZmCOP1a* and *ZmCOP1b* mutants and transgenic lines.

Figure S3 ZmCOP1a and ZmCOP1b promote seedling etiolation in Arabidopsis.

Figure S4 Phenotypes of inbred line B73 (wild type 1, WT1), inbred line B104 (transgenic background, WT2), single mutants, double mutant and overexpression lines of *ZmCOP1a* and *ZmCOP1b* under far-red (FR), red (R), or blue (B) light conditions.

Figure S5 Both *ZmCOP1a* and *ZmCOP1b* transgenic lines reduce plant height and ear height in maize

Figure S6 Molecular and biochemical evidences underlying ZmCOP1a/b-mediated plant height reduction.

Figure S7 Both ZmCOP1a and ZmCOP1b delay flowering in Arabidopsis.

Figure S8 Interactions between different functional domains of ZmCOP1 with ZmCOL3 in yeast.

Figure S9 ZmCOL3 delays flowering under LD in maize.

Figure S10 *ZmCOP1a*/*ZmCOP1b* and *ZmCOL3* mutually enhance each other’s transcription in maize.

Figure S11 Proposed regulatory module of ZmCOP1s–ZmCOL3 in controlling maize flowering time.

Figure S12 Effect of proteasome inhibitor MG132 on ZmCOL3 protein accumulation.

Figure S13 Differential gene expression in the *zmcop1a*, *zmcop1b*, and *ZmCOL3-OE* at the V6 stage.

Figure S14 ZmCOL3 modulates kernel development, starch-protein composition, and yield-related traits.

Figure S15 Phenotypic analysis of yield-related traits in *zmcop1a/zmcol3* and *zmcop1b/zmcol3* double mutants.

Figure S16 Expression analysis of zein genes in 15-DAP kernels by RT-qPCR.

Table S1 Statistics of the sequencing reads and alignment results in RNA-seq.

Table S2 Differentially expressed genes (DEGs) in *zmcop1a*, *zmcop1b*, and Z*mCOL3-OE*.

Table S3 Gene ontology (GO) enrichment analyses of DEGs in *zmcop1a*, *zmcop1b*, and *ZmCOL3-OE*.

Table S4 Kyoto Encyclopedia of Genes and Genomes (KEGG) pathway analyses of DEGs in *zmcop1a*, *zmcop1b*, and *ZmCOL3-OE*.

Table S5 Comparison of grain size and yield characteristics.

Table S6 Primers used in this study.

**
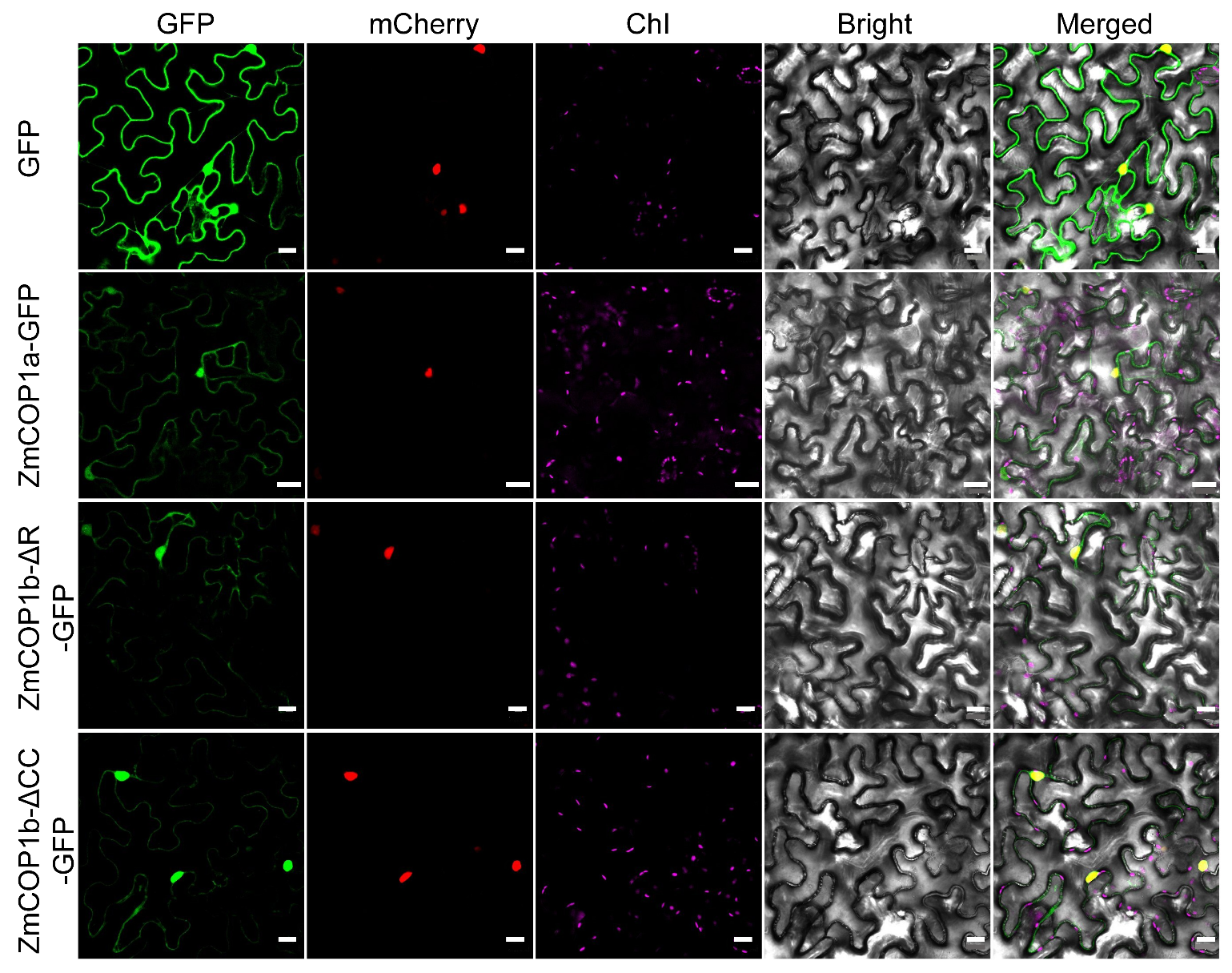
**

**Figure S1 Subcellular localization of ZmCOP1a and ZmCOP1b in tobacco leaf epidermal cells.** ZmCOP1b-ΔR denotes a ZmCOP1b variant lacking the RING finger domain (R); ZmCOP1b-ΔCC indicates a variant without the coiled-coil (CC) domain. Scale bar = 50 µm. (Related to Figure 1)


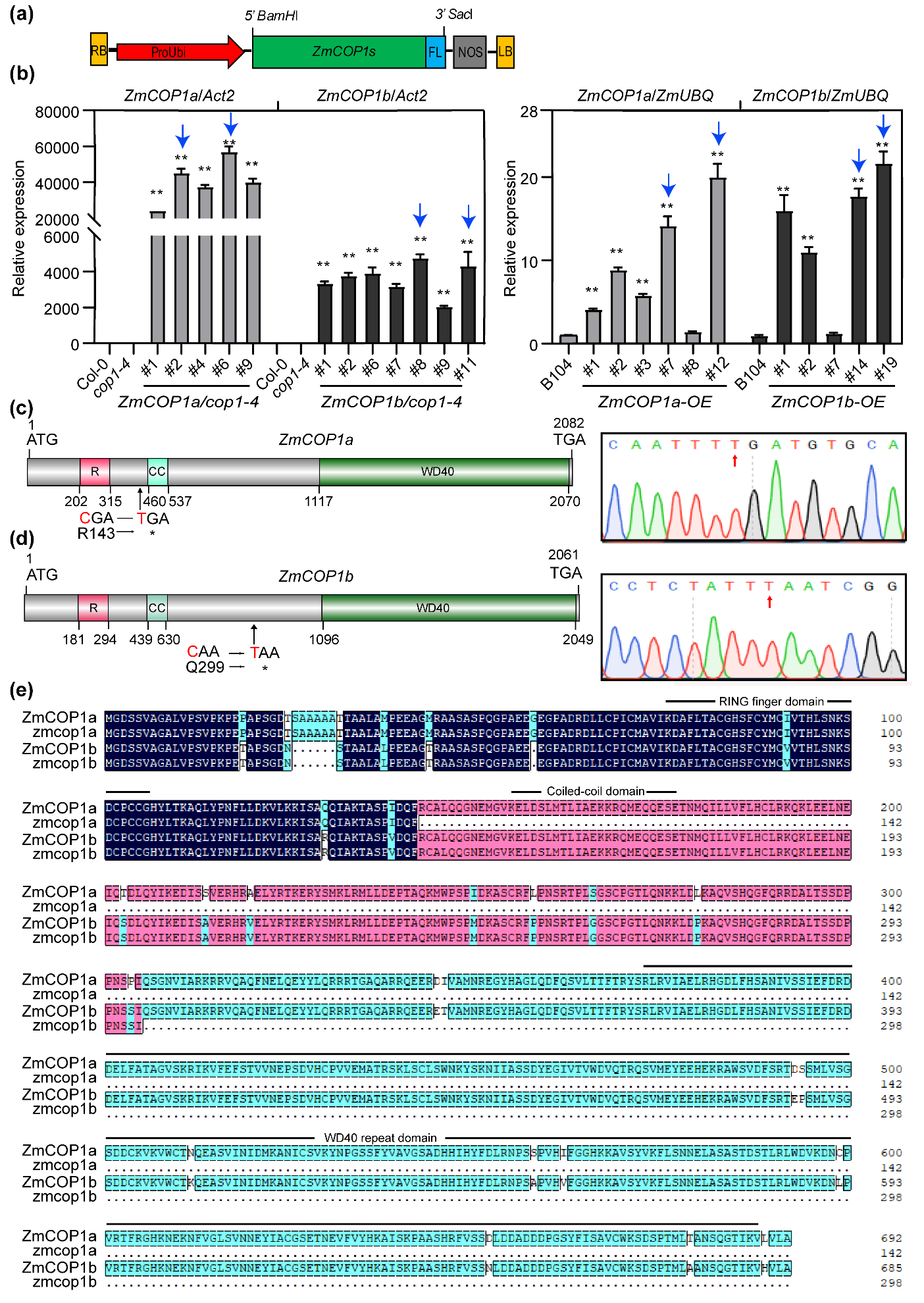


**Figure S2 Identification of *ZmCOP1a* and *ZmCOP1b* mutants and transgenic lines.** **(a)** Schematic diagrams of the binary vectors used for *ZmCOP1a* and *ZmCOP1b* transformation. FL, 3×Flag tag. **(b)** Relative expression levels of ZmCOP1a and ZmCOP1b in transgenic *Arabidopsis* and maize lines. Samples were collected from 25-day-old *Arabidopsis* seedlings or the third leaf of maize plants at the V6-stage. Asterisks (**) indicate significant differences at *P < 0.01*. Blue arrows mark the lines chosen for subsequent studies. **(c, d)** Genomic structures and mutation sites of *ZmCOP1a* (c) and *ZmCOP1b* (d). Functional domains are annotated: R, RING finger; CC, coiled-coil; WD40, WD40 repeat domain. Asterisks (*) indicate introduced stop codons; red arrows mark the sites of nucleotide changes. **(e)** Alignment of amino acid sequences between ZmCOP1s and AtCOP1. (Related to Figure 2)


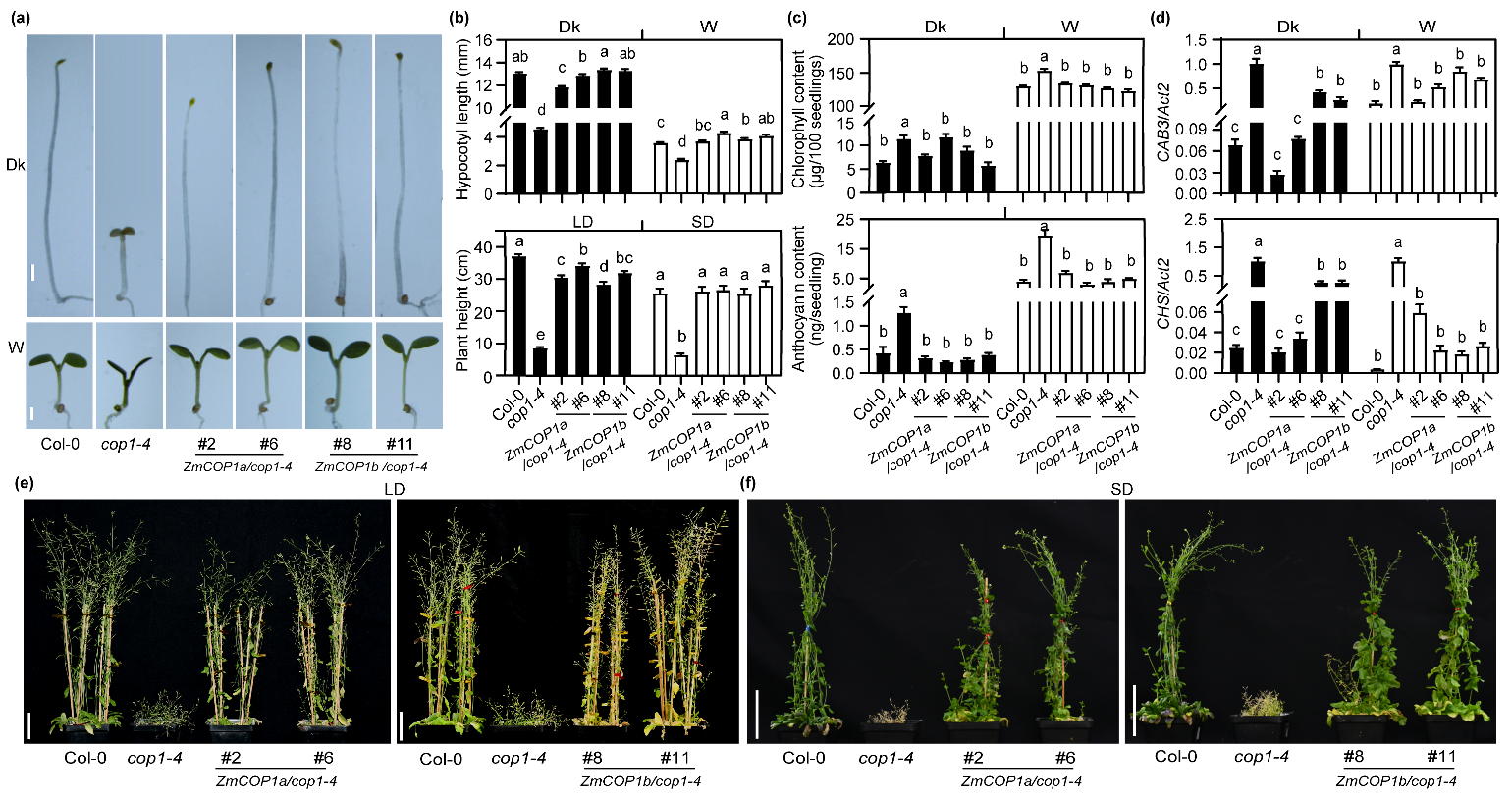


**Figure S3 ZmCOP1a and ZmCOP1b promote seedling etiolation in *Arabidopsis*. (a)** Morphology of Col-0 (wild type), *cop1-4*, *ZmCOP1a*/*cop1-4*, and *ZmCOP1b*/*cop1-4* (in *cop1-4* background) grown for 5 days at 22 °C under darkness (Dk) or white light (W, 17 μmol·m⁻²·s⁻¹). Scale bar = 1 mm. **(b, c)** Hypocotyl lengths and plant heights (b, n > 40) and chlorophyll and anthocyanin contents (c) in the indicated genotypes corresponding to (a). **(d)** Relative expression levels of *CAB3* and *CHS* in the indicated genotypes corresponding to (a). Data represent mean ± SE (n = 3). **(e-f)** Morphology of adult plants of Col-0, *cop1-4*, *ZmCOP1a*/*cop1-4*, and *ZmCOP1b*/*cop1-4* grown in long-day (LD, 16 h light/8 h darkness) for 77 days or short-day (SD, 8 h light/16 h darkness) for 124 days. Scale bar = 5 cm. (Related to Figure 2)


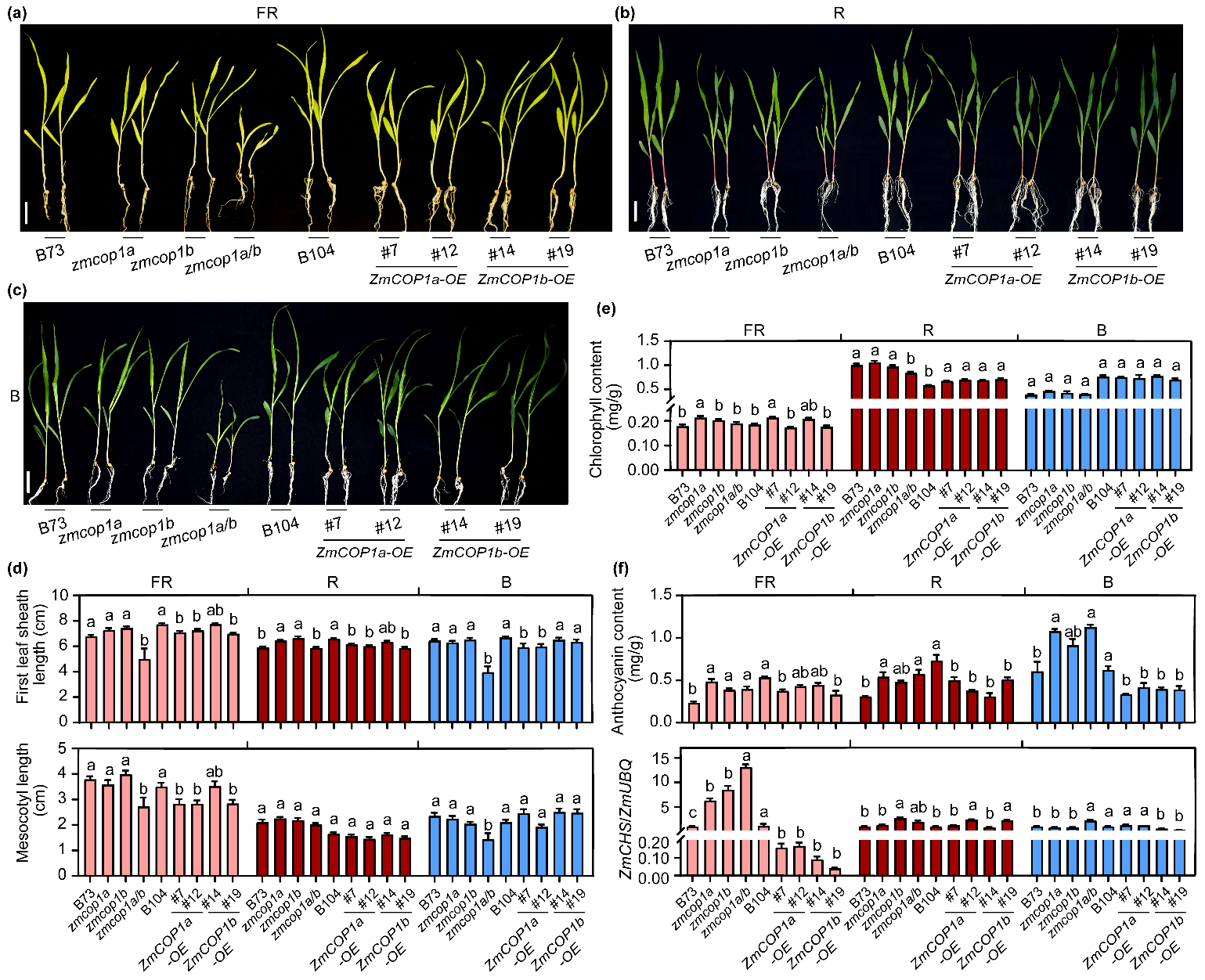


**Figure S4** **Phenotypes of inbred line B73 (wild type 1, WT1), inbred line B104 (transgenic background, WT2), single** **mutants, double mutant and overexpression lines of ZmCOP1a and ZmCOP1b under far-red (FR), red (R), or blue (B) light conditions.** Seedling were grown under FR (2.5 μmol·m⁻²·s⁻¹), R (26.0 μmol·m⁻²·s⁻¹), or B (26.0 μmol·m⁻²·s⁻¹) light at 26°C for 10 days. **(a-c)** Morphology of the indicated genotypes under FR (a), R (b), and B (c) light. Scale bar = 5 cm. **(d-f)** Measurements of the first leaf sheath and mesocotyl lengths (d), chlorophyll content (e), and anthocyanin content along with relative expression levels of *ZmCHS* (f) in seedlings shown in (a-c). Data are presented as mean ± SE (n = 3). (Related to Figure 2)


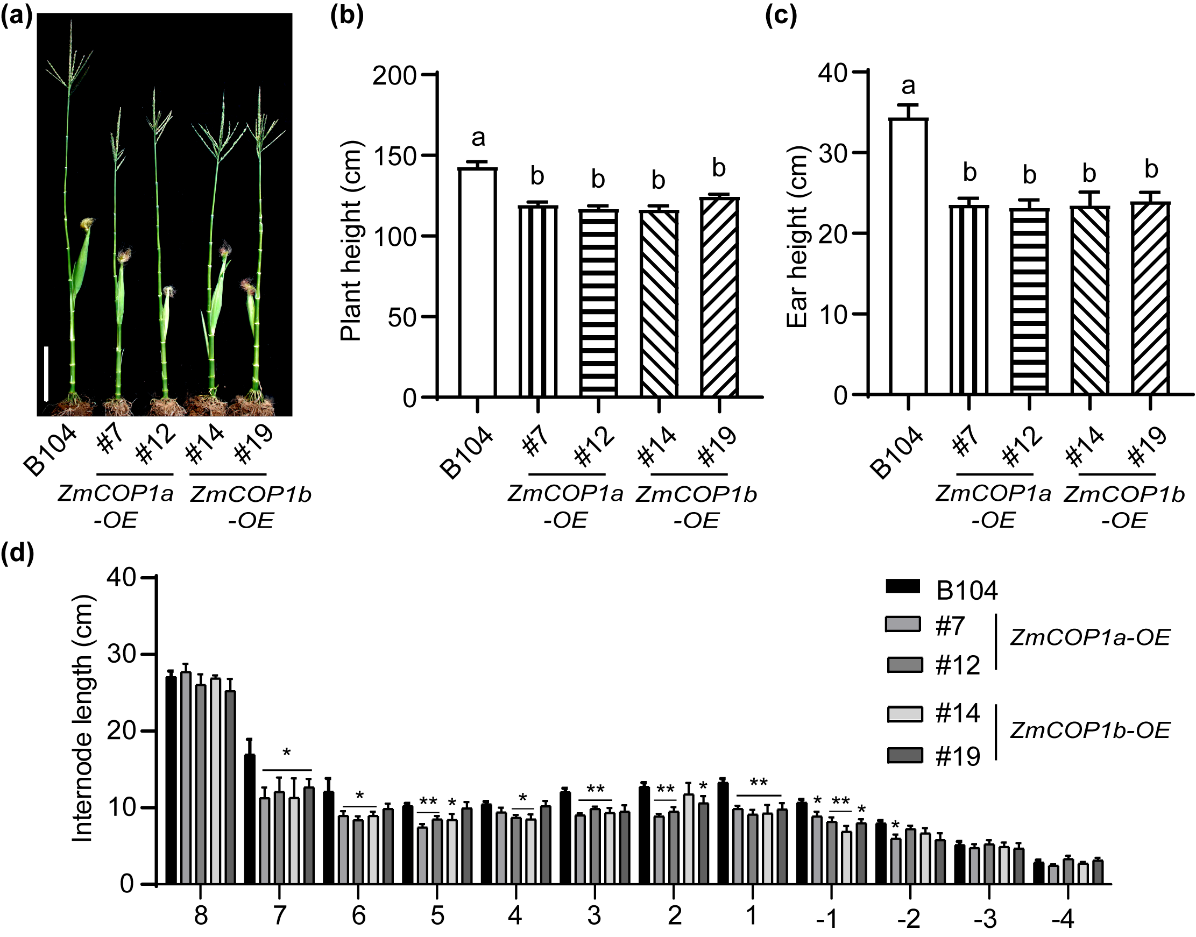


**Figure S5 Both *ZmCOP1a* and *ZmCOP1b* transgenic lines reduce plant height and ear height in maize.** **(a)** Morphology of adult B104 and *ZmCOP1a*/*b* overexpression plants grown under long-day (LD) conditions for 63 days in 2024. Scale bar = 20 cm. Plant height **(b)**, ear height **(c)** and inter-node length **(d)** measurements corresponding to (a). n ≥ 6. The ear node is marked as the first section, with the sections above the ear node being positive and those below it being negative. Different letters indicate significant differences (*P* < 0.05, one-way ANOVA followed by Turkey’s test) in (b) and (c). Data analysis of (d) using Student’s *t*-test, **P* < 0.05, ***P* < 0.01. (Related to Figure 2)


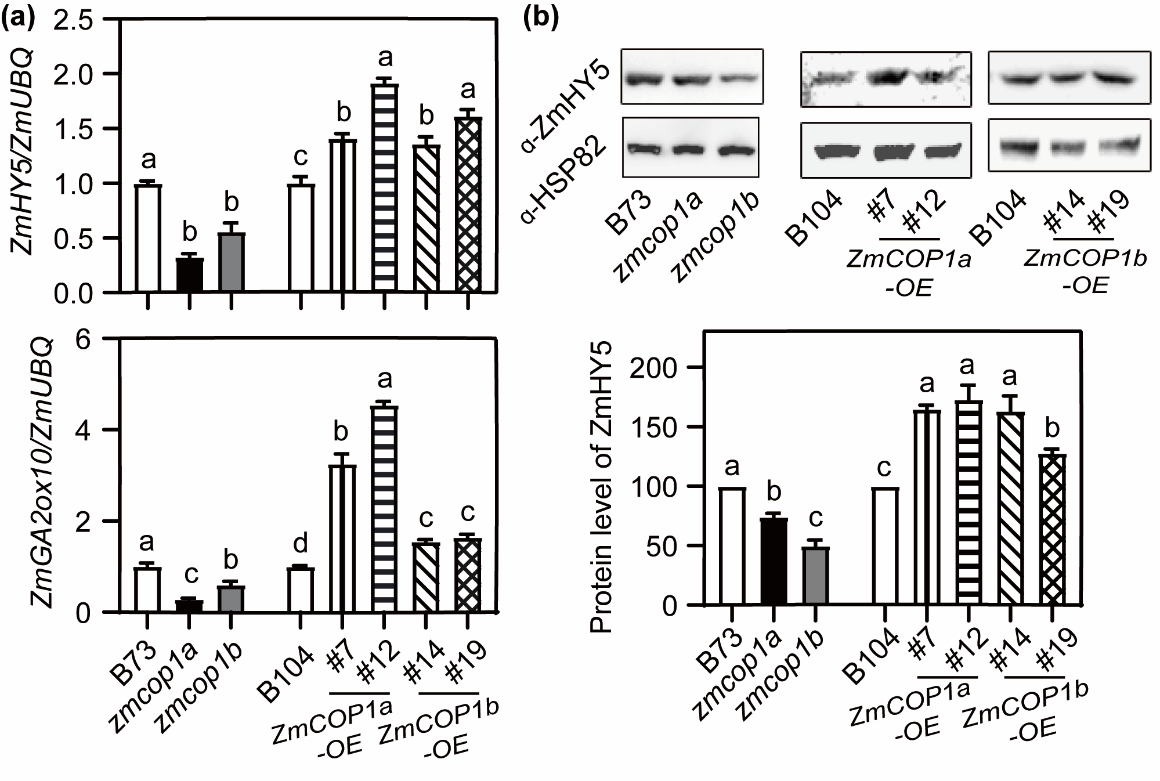


**Figure S****6 Molecular and biochemical** **evidences underlying ZmCOP1a/b-mediated plant height reduction.** The third-leaf of V6-stage seedlings were collected under LD for RNA and protein extraction. HSP82 was used as an internal control. Data represent mean ± SE (n = 3). (Related to Figure 2)


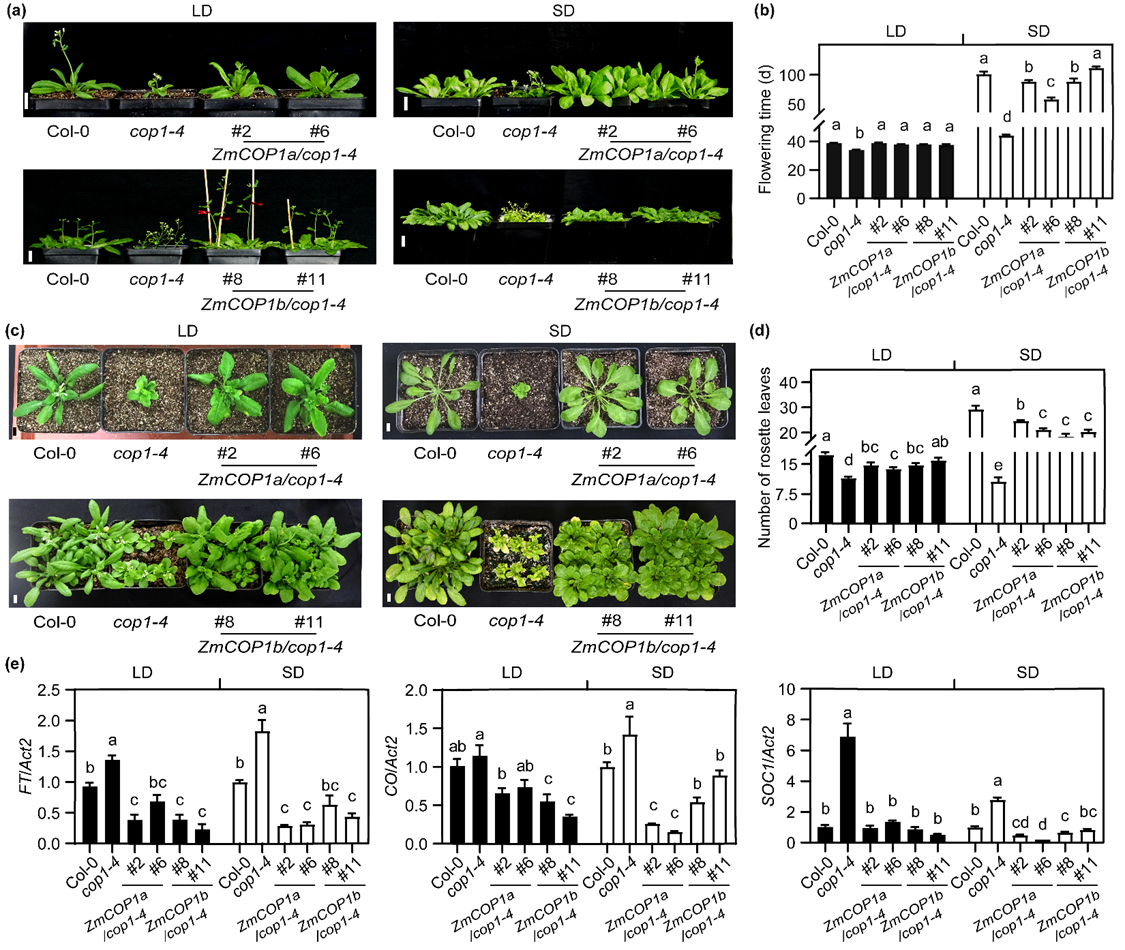


**Figure S7 Both ZmCOP1a and ZmCOP1b** **delay flowering in *Arabidopsis.* (a-d)** Flowering morphology (a), flowering times (b), rosette leaf morphology (c), and rosette leaf numbers (d) of Col-0, *cop1-4*, *ZmCOP1a*/*cop1-4* (#2, #6) and *ZmCOP1b*/*cop1-4* (#8, #11) under LD for 34 days or SD for 46 days. Scale bar = 2 cm (a), Scale bar = 1 cm (c). n > 30 (b). n > 15 (d). **(e)** Expression levels of flowering-related genes, *FLOWERING LOCUS T* (*FT*), *CONSTANS* (*CO*), and *SUPPRESSOR OF OVEREXPRESSION OF CONSTANS1* (*SOC1*) in the indicated genotypes grown under LD for 10 days or SD for 30 days. n = 3. (Related to Figure 3)


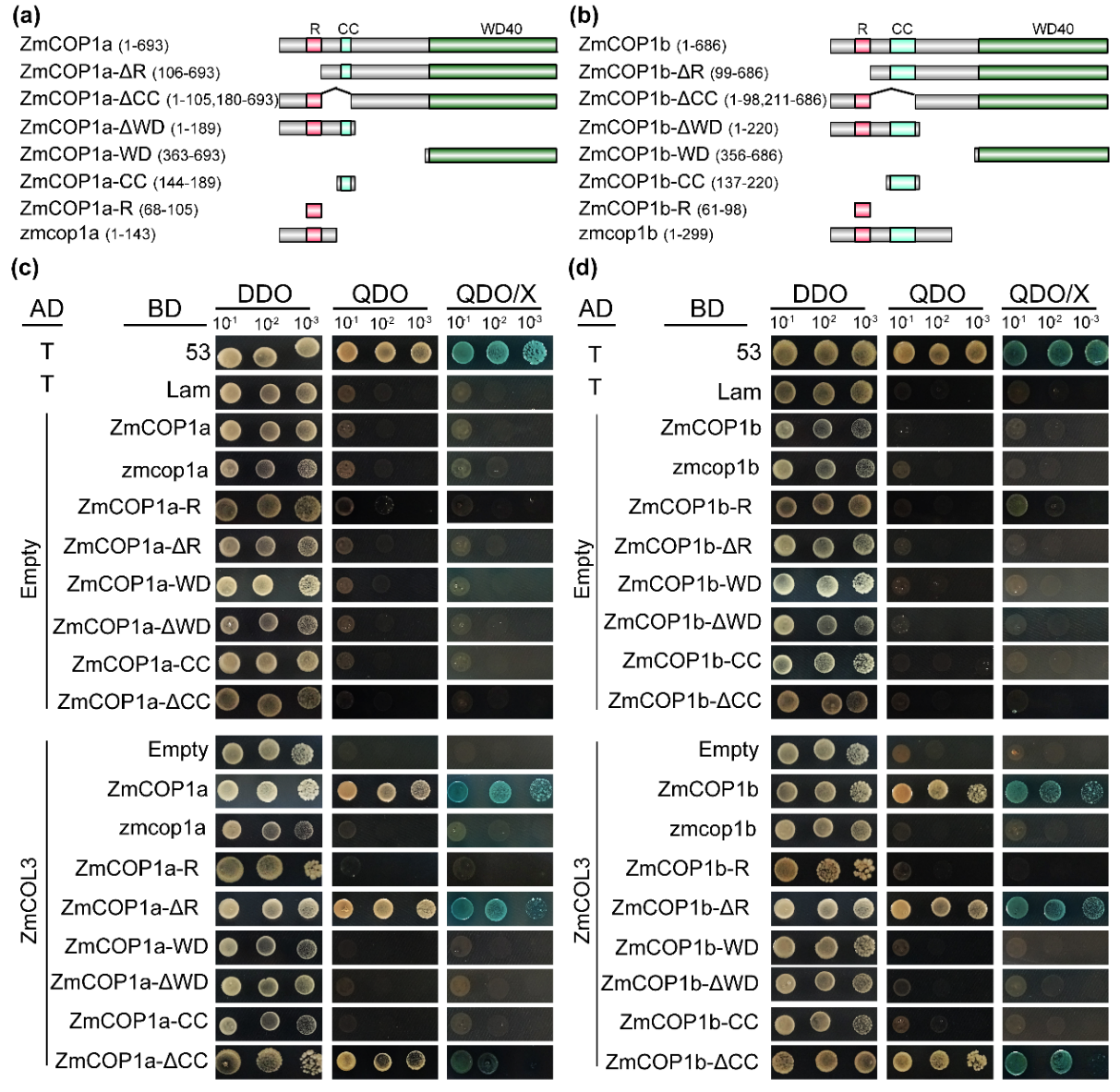


**Figure S8 Interactions between** **different functional domains of ZmCOP1 with ZmCOL3 in yeast. (a, b)** Schematic diagrams of different functional domains of ZmCOP1a (a) and ZmCOP1b (b) used in yeast two-hybrid assays (Y2H). Numbers indicate amino acid positions. **(c, d)** Y2H analysis of the interaction between ZmCOL3 with ZmCOP1a/b-ΔR or ZmCOP1a/b-ΔCC. The AD-T + BD-Lam and AD-T + BD-53 pairs were used as a negative or positive control. (Related to Figure 4)

**
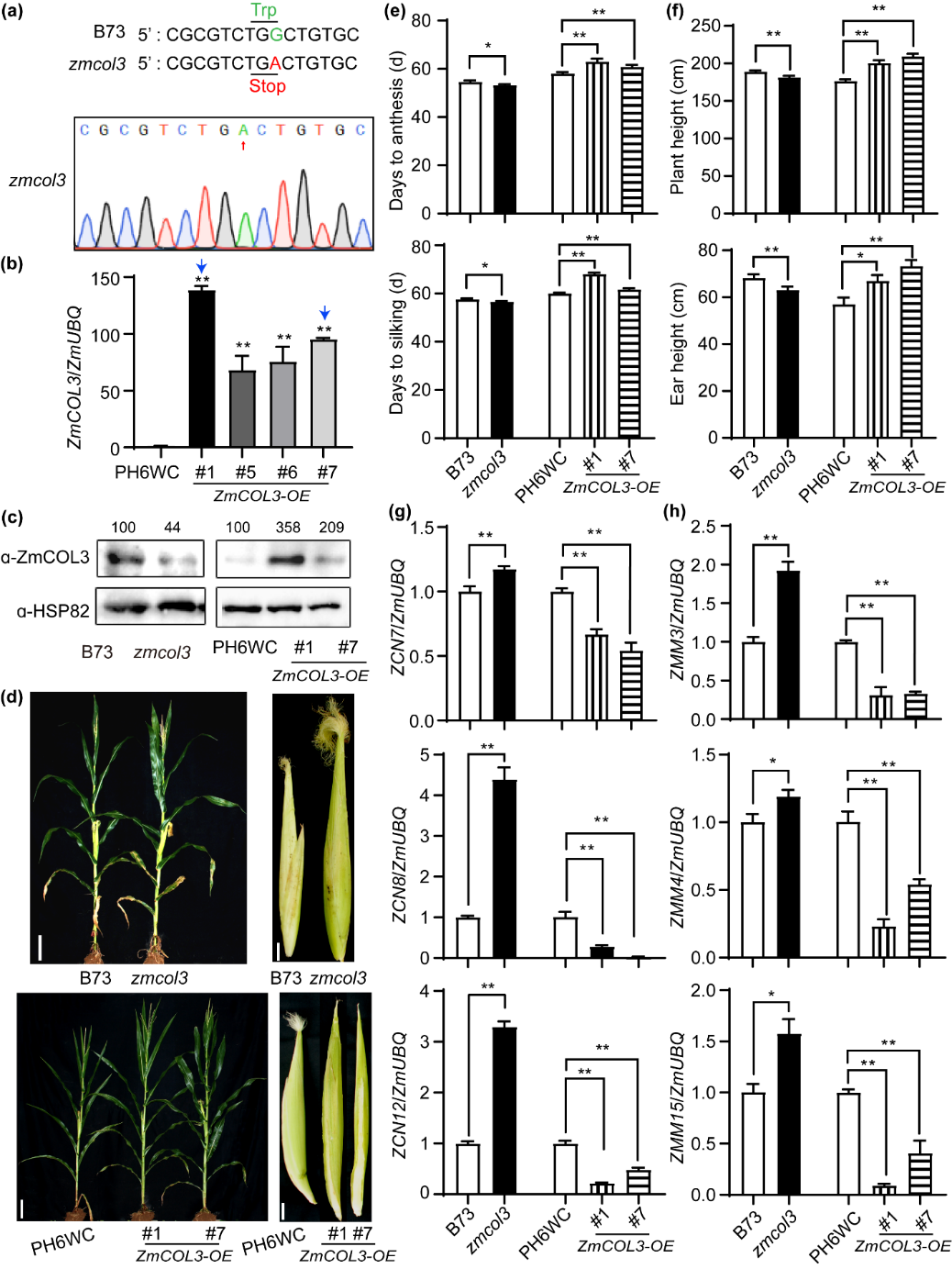
**

**Figure S9 ZmCOL3 delays flowering under LD in maize. (a)** Identification of the *zmcol3* mutant allele. The red arrow indicates the mutation site. **(b)** Screening and selection of *ZmCOL3* transgenic lines. Blue arrows mark the lines chosen for further analysis. **(c)** Immunoblot detection of ZmCOL3 protein levels in mutant and transgenic materials, using HSP82 as a loading control. **(d)** Plant and ear phenotypes of the mutant and overexpression lines of *ZmCOL3* at the pollination stage. Scale bars: 20 cm (plant), 2 cm (ear). **(e)** Silking and anthesis times in the mutant and transgenic lines of *ZmCOL3*. **(f)** Plant height and ear height of the indicated genotypes. Mutants, n ≥ 10; transgenic lines, n ≥ 15. **(g, h)** Expression levels of flowering-time genes in the mutant and transgenic lines of *ZmCOL3*. **P* < 0.05, ***P* < 0.01. (Related to Figure 4)


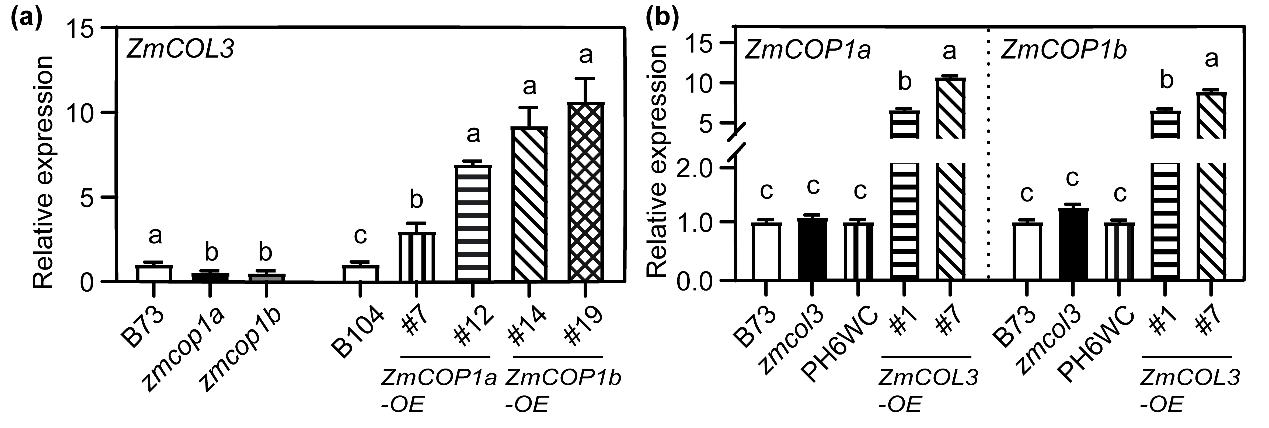


**Figure S10** ***ZmCOP1a*/*ZmCOP1b* and *ZmCOL3* mutually enhance each other’s transcription in maize.** Samples were collected from the third leaf of maize plants at the V6 stage under LD. **(a)** Relative expression level of *ZmCOL3* in the mutants and transgenic lines of *ZmCOP1a*/*ZmCOP1b*. **(b)** Relative expression level of *ZmCOP1a* and *ZmCOP1b* in the mutant and transgenic lines of *ZmCOL3*. Data represent mean ± SE (n = 3). (Related to Figure 4)


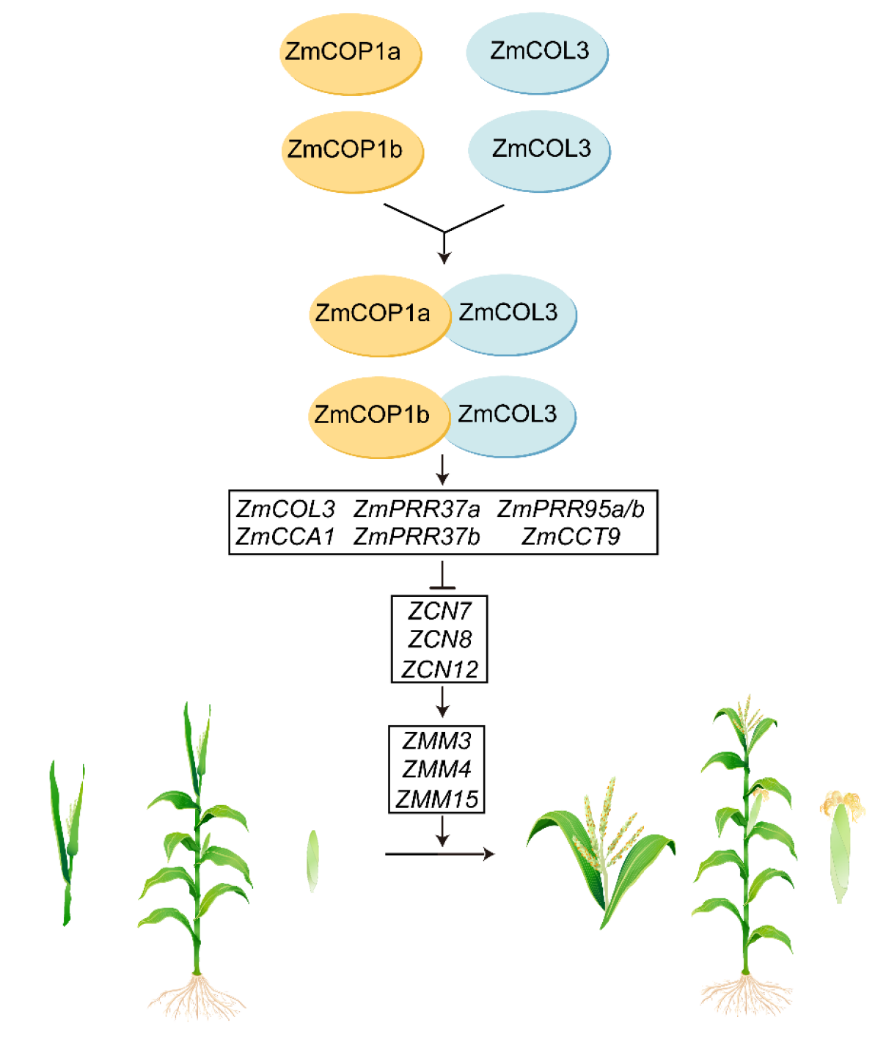


**Figure S11 Proposed regulatory module of ZmCOP1s–ZmCOL3 in controlling maize flowering time.** ZmCOP1a and ZmCOP1b, containing RING finger (R), coiled-coil (CC), and WD40 repeat domains, interact with ZmCOL3 to form ZmCOP1s-ZmCOL3 complex. This complex promotes the expression of photoperiod-related repressors (*ZmPRR37a*/*b*, *ZmPRR95a*/*b*, *ZmCCA1*, and *ZmCCT9*) and upregulates *ZmCOL3*, while repressing florigen genes (*ZCN7*/*8*/*12*) and flowering markers (*ZMM3*/*4*/*15*), ultimately delaying silking and flowering. (Related to Figure 4)


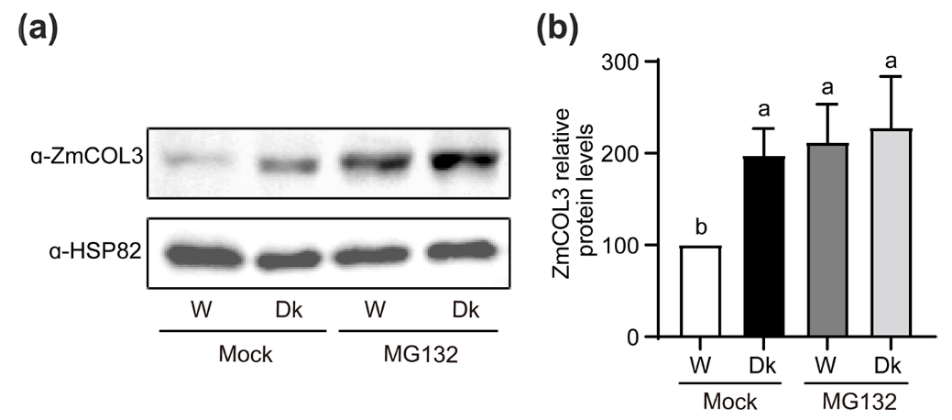


**Figure S12 Effect of proteasome inhibitor MG132 on ZmCOL3 protein accumulation.** Seedlings of maize inbred line B73 were grown under white light (70 μmol·m⁻²·s⁻¹) at 26°C for 10 days. To investigate ubiquitin-mediated protein degradation, seedlings were treated with 7 μM MG132 (a 26S proteasome inhibitor) for 14 hours under either darkness or white light (70 μmol·m⁻²·s⁻¹) at 26°C. Leaves were then harvested for protein extraction. HSP82 served as the internal control. (Related to Figure 4)


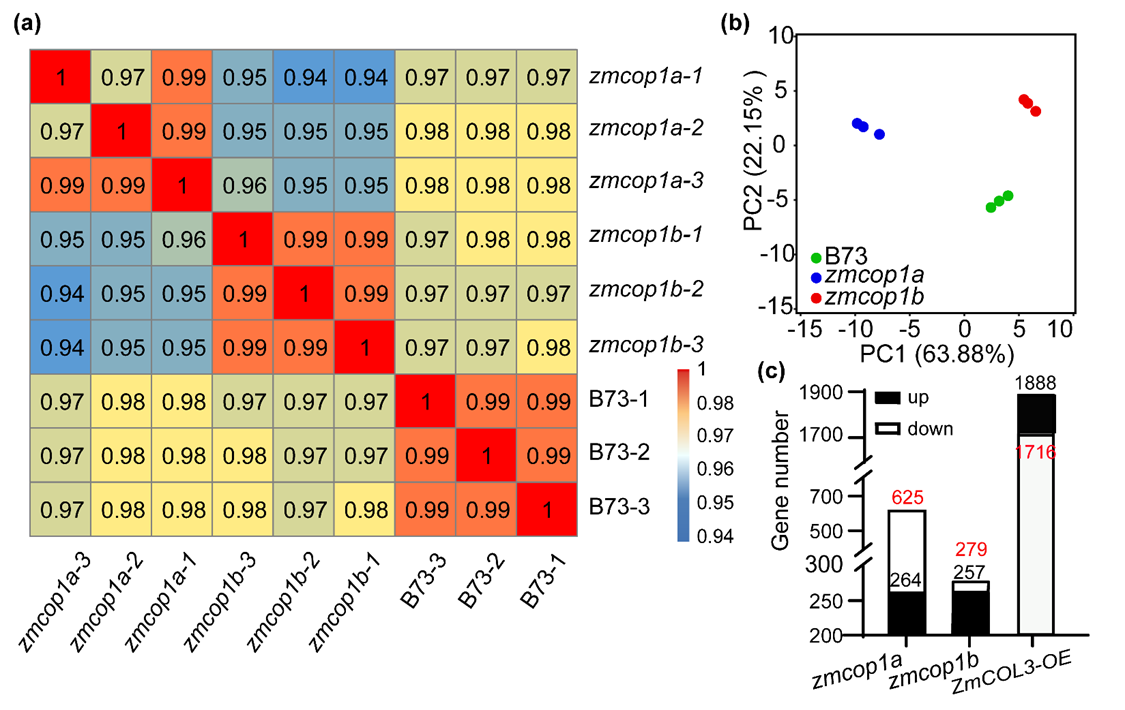


**Figure S13 Differential gene expression in the *zmcop1a*, *zmcop1b*, and *ZmCOL3-OE* at the V6 stage. (a)** Correlation coefficients (R) among three biological replicates. **(b)** Principal component analysis (PCA) of transcriptome profiles. **(c)** Numbers of up- and down-regulated differentially expressed genes (DEGs) in each genotype (fold change > 2, *P < 0.05*). (Related to Figure 5)


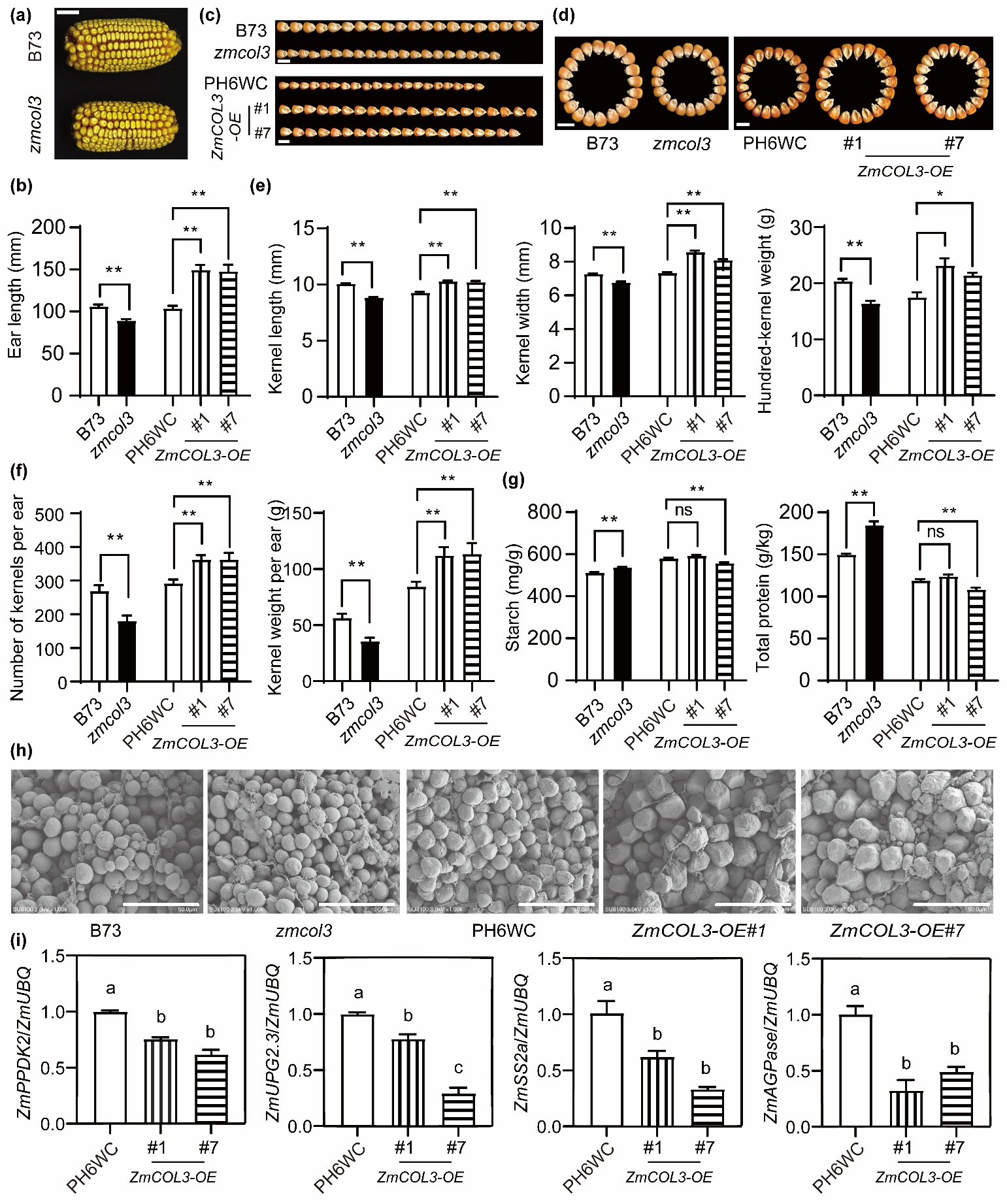


**Figure S14 ZmCOL3 modulates kernel development, starch-protein composition, and yield-related traits. (a)** Ear phenotype of the *zmcol3* mutant. Scale bar = 2 cm. **(b)** Ear length in the mutant and transgenic lines of *ZmCOL3* (n ≥ 10). **(c–e)** Kernel length, kernel width, and hundred-kernel weight of the mutant and transgenic lines of *ZmCOL3*. Scale bar = 1 cm. **(f)** Kernel number per ear and total kernel weight per ear (n ≥ 10). **(g)** Starch and total protein content in mature kernels. Data represent mean ± SE (n = 3). **(h)** Scanning electron micrographs of mature kernel endosperm. Scale bar = 50 μm. **(i)** Expression analysis by RT–qPCR of starch metabolic genes in V6 leaves. Data are shown as mean ± SE (n = 3). (Related to Figure 6)


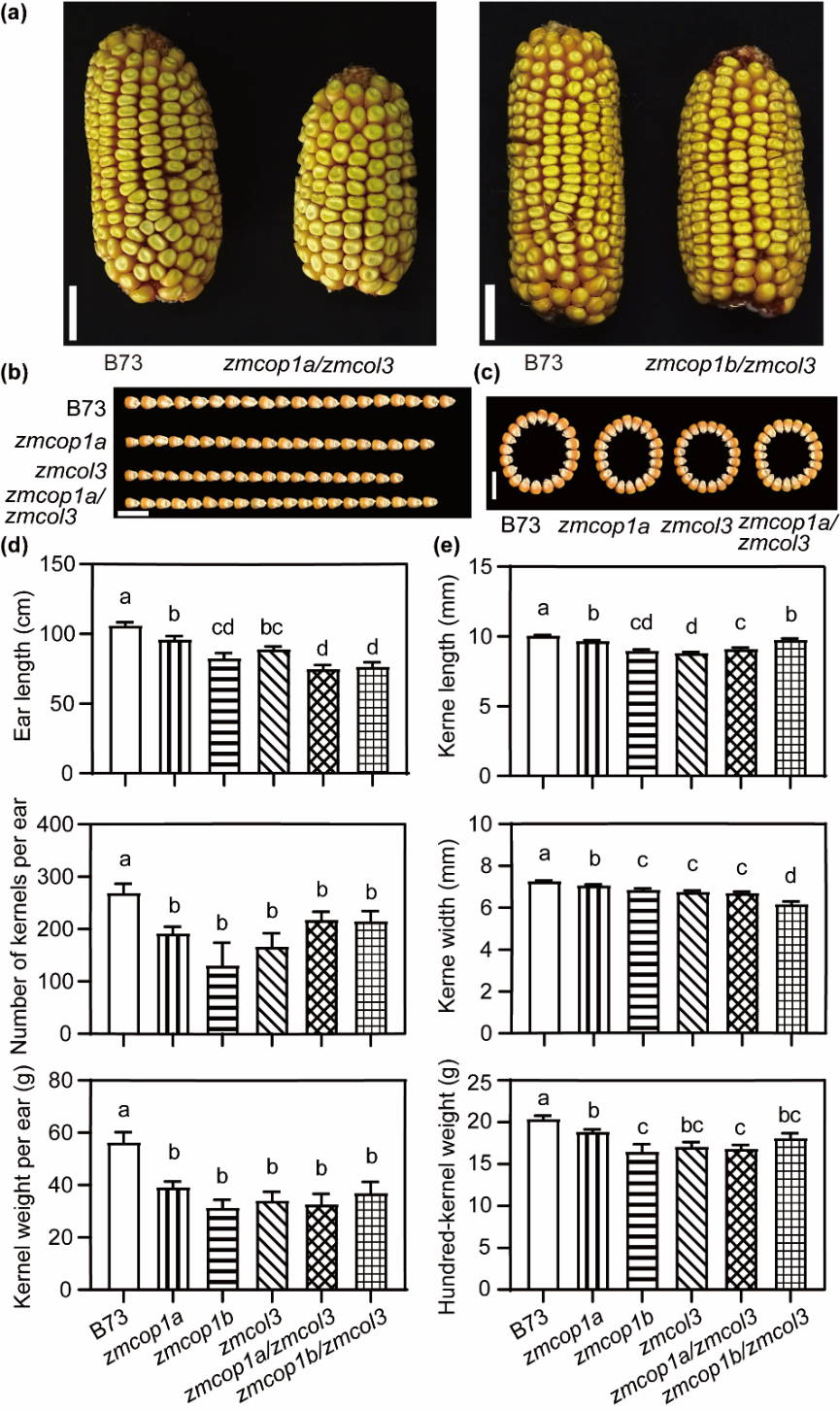


**Figure S15 Phenotypic analysis of yield-related traits in *zmcop1a*/*zmcol3* and *zmcop1b*/*zmcol3* double mutants. (a-c)** Morphological phenotypes of mature ear, kernel length, and kernel width in *zmcop1a/zmcol3* and *zmcop1b*/*zmcol3* double mutants. Scale bar = 2 cm (a) and 1 cm (b-c). **(d)** Quantitative analysis of ear length, kernel number per ear, and kernel weight per ear as shown in (a) (n ≥ 9). **(e)** Quantitative analysis of kernel length, kernel width, and hundred-kernel weight as shown in (b, c) (n ≥ 38). Data represent mean ± SE (n = 3). (Related to Figure 6).


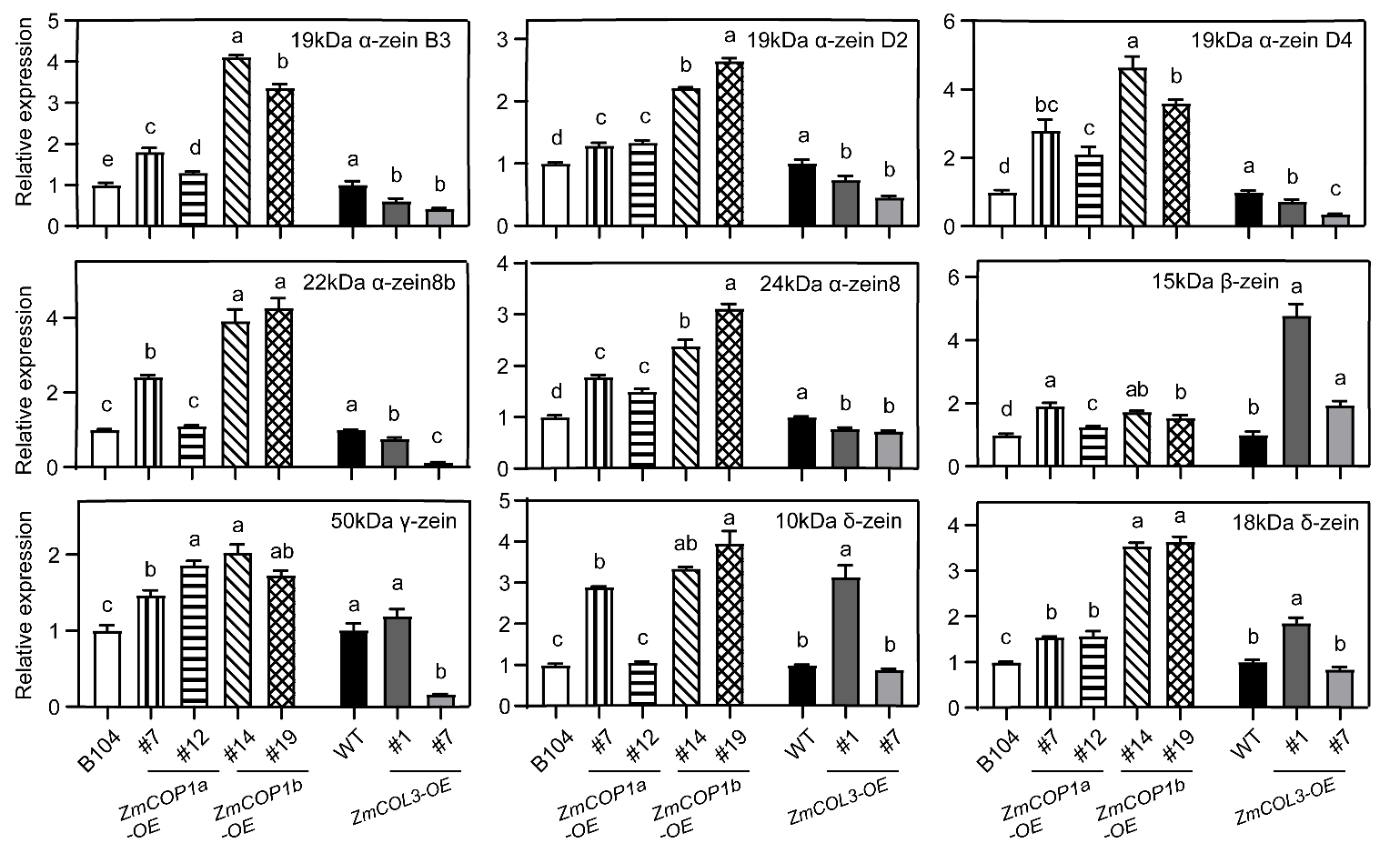


**Figure S16 Expression analysis of zein genes in 15-DAP kernels by RT-qPCR.** Data represent mean ± SE (n = 3). (Related to Figure 6)
